# Observation of directional leaky polaritons at anisotropic crystal interfaces

**DOI:** 10.1038/s41467-023-38326-7

**Published:** 2023-05-18

**Authors:** Xiang Ni, Giulia Carini, Weiliang Ma, Enrico Maria Renzi, Emanuele Galiffi, Sören Wasserroth, Martin Wolf, Peining Li, Alexander Paarmann, Andrea Alù

**Affiliations:** 1 Photonics Initiative, Advanced Science Research Center, City University of New York, New York, NY 10031 USA; 2 School of Physics and Electronics, Central South University, Changsha, Hunan 410083 China; 3 Fritz Haber Institute of the Max Planck Society, Berlin, Germany; 4 School of Optical and Electronic Information, Wuhan National Laboratory for Optoelectronics and Wuhan National high Magnetic Field Center, Huazhong University of Science and Technology, Wuhan, China; 5 Optics Valley Laboratory, Hubei, 430074 China; 6 Physics Program, Graduate Center, City University of New York, New York, NY 10016 USA

**Keywords:** Nanophotonics and plasmonics, Sub-wavelength optics

## Abstract

Extreme anisotropy in some polaritonic materials enables light propagation with a hyperbolic dispersion, leading to enhanced light-matter interactions and directional transport. However, these features are typically associated with large momenta that make them sensitive to loss and poorly accessible from far-field, being bound to the material interface or volume-confined in thin films. Here, we demonstrate a new form of directional polaritons, leaky in nature and featuring lenticular dispersion contours that are neither elliptical nor hyperbolic. We show that these interface modes are strongly hybridized with propagating bulk states, sustaining directional, long-range, sub-diffractive propagation at the interface. We observe these features using polariton spectroscopy, far-field probing and near-field imaging, revealing their peculiar dispersion, and – despite their leaky nature – long modal lifetime. Our leaky polaritons (LPs) nontrivially merge sub-diffractive polaritonics with diffractive photonics onto a unified platform, unveiling opportunities that stem from the interplay of extreme anisotropic responses and radiation leakage.

## Abstract (web summary)

A new form of directional polaritons, leaky in nature and featuring lenticular dispersion contours, is experimentally observed both in near-field and through prism excitation, unveiling opportunities stemming from the interplay of extreme anisotropic responses, light confinement and directional radiation leakage.

## Introduction

Several natural materials, including van der Waals materials, semiconductors, and dielectric polar crystals, have been attracting significant attention in recent years for their structural, electronic, and optical properties, forming an excellent platform to engage strong interactions between light and dipole-active material excitations^1–5^. Their strong coupling with light supports the emergence of exotic half-light half-matter particles, known as polaritons, across a broad range of wavelengths from the mid-infrared to the visible and ultraviolet regimes. When polaritonic materials have principal elements of their permittivity tensor with opposite signs, they support hyperbolic modes, recently observed in a variety of natural materials, including graphite^6^, tetradymite (Bi_2_Te_2_S)^7^, hexagonal boron nitride (h-BN)^8^, alpha-phase molybdenum trioxide (α-MoO_3_)^9^, alpha-phase vanadium pentoxide (α-V_2_O_5_)^10^, and hybrid phonon-plasmon polaritonic materials^11,12^. Their extreme optical anisotropy, stemming from exciton or phonon resonances polarized in specific directions as a function of the underlying lattice geometry, enables enhanced light-matter interactions, nanoscale localization of electromagnetic energy, and highly directional propagation beyond the diffraction limits. These features are directly associated with the open topology of their hyperbolic dispersion contours^6^. In h-BN, such extreme anisotropy is observed with respect to the materials interface normal, implying that hyperbolic polaritons travel in the bulk, making their direct observation and manipulation challenging^8,13–19^. On the contrary, α-MoO_3_ features a lattice configuration supporting extreme anisotropy in the interface plane, leading to hyperbolic polaritons in thin films, more easily accessible through nanoscale objects close to the interface with air^9,20–24^, and providing opportunities for twisted nano-optics^25–28^. Calcite (calcium carbonate)^29,30^, as another remarkable example, features extreme anisotropy whose optical axis (OA) orientation can be controlled with respect to the material interface with air, leading to ghost hyperbolic polaritons (g-HPs) bound to the interface but with tilted phase fronts entering the bulk^31^. Further lowering the symmetry, monoclinic crystals like β-Ga_2_O_3_ have been recently shown to support hyperbolic shear polaritons, in which non-orthogonal phonon resonances sustain hyperbolic propagation with asymmetric loss profiles and an OA rotating as a function of frequency^32^. The growing family of polaritonic materials has been opening unique opportunities for light-matter nanoscale routing, transport, and manipulation.

So far, all polaritons and the associated exciting features have been strictly bound to the material they live in, either in the form of volume-confined modes or of surface waves traveling along the interface planes, evanescently decaying away from the surface and exhibiting sub-diffractive features^33^. In the limit of negligible material loss, these guided modes feature real-valued in-plane momenta larger than the one in the surrounding materials, associated with the bound nature of their propagation. In conventional refractive optics, it is common to consider leaky waves as a different class of guided modes at an interface, featuring a complex in-plane momentum living within the light cone of at least one of the materials forming the interface and hence associated with radiation leakage^34–37^.

In this work, we merge these fields of research and demonstrate that natural anisotropic crystals can support a regime for polariton propagation inherently leaky in nature, which is at the same time bound to the interface with air and hybridized with refractive bulk modes. This class of polaritons features a form of iso-frequency contours (IFCs) of dispersion with a peculiar lenticular shape, forming a cusp or a wedge at a high-symmetry point in momentum space. Interestingly, despite featuring IFCs with a closed topology, i.e., limited to a finite region in momentum space, these leaky polaritons (LPs) support long-distance and directional propagation along the material interface with large Q-factors. These features are remarkably different from conventional leaky-waves.

## Results

### Eigenmode analysis with complex-valued wavenumber

**Fig. 1:**
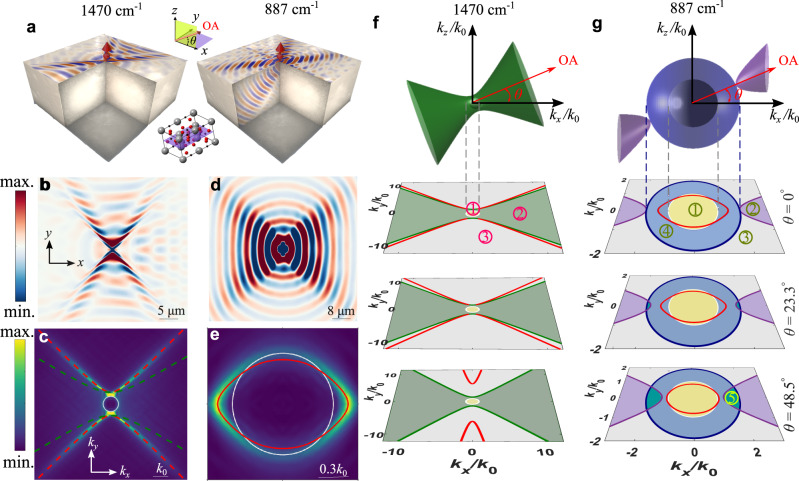
Leaky polaritons (LPs). **a** Schematic of the excitation of ghost hyperbolic polaritons (g-HPs) in the left panel and LPs in the right panel, emerging at the interface between calcite and air and supporting directional waves that propagate on the surface and in the bulk of calcite. **b**, **d** Real space distribution of simulated field ${\mathfrak{R}}({{{{{{\rm{E}}}}}}}_{z})$ on the surface of calcite and **c**, **e** the corresponding Fourier Transformation (FT) in *k*-space, respectively. **f** 3D isofrequency contours (IFCs) for type-II hyperbolic regime at frequency ($\omega=1470\,{{{{{{\rm{cm}}}}}}}^{-1}$) supporting g-HPs and **g** 3D IFCs for type-I hyperbolic regime at frequency ($\omega=887\,{{{{{{\rm{cm}}}}}}}^{-1}$) supporting LPs, and their dependence of the in-plane momentum dispersion on the slanted optical axis of calcite. The green solid lines in **f** (green dashed lines in **c**) represent the light cones of extraordinary wave in type-II hyperbolic regime, blue and purple solid lines in **g** represent the light cones of ordinary wave and extraordinary wave in the type-I hyperbolic regime, respectively. The red solid lines in **f** (red dashed lines in **c**) are the dispersion of g-HPs, while the red solid lines in **e**, **g** show the dispersion of LPs, obtained from real- and complex-valued Eq. (1), respectively. The white sphere depicts the IFC of free space, and white circle is the free space light cone (FSLC).

To reveal their features, we study the exemplary case of polaritons at the interface between air and calcite. Calcite’s OA can be slanted with respect to the interface by an angle $\theta$ [Fig. 1a]. Polaritons at the air-calcite interface have been recently explored in the upper Reststrahlen band^31^, which are associated with a type-II hyperbolic dispersion of their phonon polaritons [see material dispersion in Supplementary Information, Fig. S1]. When $\theta\, \ne \, {0}^{\circ }$, the broken symmetry between the phonons and the interface tilts the polariton’s wave vectors with respect to the interface but are still bound to the interface, leading to the emergence of g-HPs, as shown in Fig. 1a for $\omega=1470\,{{{{{{\rm{cm}}}}}}}^{-1}$. The Fourier transform (FT) of the in-plane fields (Fig. 1b) is associated with a hyperbolic IFC, shown in Fig. 1c. In contrast, the lower Reststrahlen band, for which ${\epsilon }_{\parallel }\, < \, 0$ and ${\epsilon }_{\perp }\, > \, 0$, supports type-I hyperbolic phonon polaritons in the bulk. This frequency range, as well as the neighboring ε-near-zero transparent regime where ${\epsilon }_{\perp }\, > \,0$ and $0\, < \,{\epsilon }_{\parallel }\, < \,{\epsilon }_{{{{{\rm{air}}}}}}$, both support the emergence of LPs at the interface with air. When excited by a localized emitter in the hyperbolic regime at $\omega=887{{{{{{\rm{cm}}}}}}}^{-1}$ (Fig. 1a), the LP field distribution is remarkably directional in the interface plane (Fig. 1d), showing four emission lobes. The corresponding FT, however, is associated with an IFC with a peculiar lenticular shape (Fig. 1e), with a closed topology despite the directionality of emission.

These dispersion features can be analyzed by solving source-free Maxwell’s equations at the air-calcite interface, for the moment neglecting material absorption. The polariton dispersion can be computed by enforcing the continuity of the tangential fields at the interface: we generally assume that the in-plane wavenumbers in the *x*/*y* directions ${k}_{x,y}$ are real-valued, and obtain

1 $$\det (\widehat{{{{{{\rm{D}}}}}}}({k}_{x},\, {k}_{y}))=0,$$

where $\hat{{{{{{\rm{D}}}}}}}$ is a $4\times 4$ matrix (see Supplementary Information Section I). The in-plane dispersion of g-HPs in the type-II hyperbolic regime at $\omega=1470\,{{{{{{\rm{cm}}}}}}}^{-1}$ is shown as a red dashed curve in Fig. 1c, in agreement with the FT of the near-fields. In this regime, calcite only supports the propagation of extraordinary waves in bulk, and their IFC is a hyperboloid aligned with the OA (generally oriented at an angle $\theta$ with respect to the interface, as shown in Fig. 1f). Around the origin, we also show the spherical IFC of free-space radiation modes. When projected onto the interface plane, three different regions emerge for the in-plane momentum: (1) the free space light cone (FSLC) (white circle), (2) the light cone of extraordinary waves in calcite (green hyperbolae), and (3) the remainder of the in-plane *k*-space (gray) where no bulk modes are supported at either side of the interface. Surface hyperbolic polaritons (s-HPs) $(\theta={0}^{\circ })$ and g-HPs ($\theta\, \ne \,{0}^{\circ }$) guided at the interface and with real in-plane wave numbers are supported in the region (3), with their dispersion depicted by red lines in Fig. 1f, bound to the interface, carrying power along it and evanescently decaying away from it.

In contrast, LPs result from the hybridization of surface-bound modes with propagating bulk waves. They naturally emerge in the type-I hyperbolic regime, e.g., at $\omega=887\,{{{{{{\rm{cm}}}}}}}^{-1}$ for calcite, in which bulk propagation is now associated with ordinary waves with isotropic dispersion in *k*-space, described by the outer sphere in Fig. 1g (top panel), as well as extraordinary waves with hyperbolic dispersion, described by the two purple hyperboloids with major axis parallel to the OA. The projected dispersions onto the *k*_*x*_–*k*_*y*_(interface) plane show a more complex geometry, with four distinct regions: regions (1–3) (R1–R3) resemble the ones in the type-II hyperbolic regime. In addition, R(4) outside the FSLC (blue ring) supports the propagation of ordinary bulk waves. Thus, while the extraordinary interface mode with in-plane momentum in R(1) can radiatively leak to both sides of the interface, in R(4) the interface modes are confined on the air side but leak into a refractive ordinary wave in calcite (see Supplementary Information Section I). As we tilt the OA with respect to the interface, $\theta \ne {0}^{^\circ }$ (bottom panels of Fig. 1g) a new region, R(5), appears due to the skewed hyperboloid projection overlapping with R(4), thus supporting radiation leakage into ordinary and extraordinary waves. The in-plane momentum diagram in Fig. 1g implies that the interface modes can become compatible with radiation towards both sides of the interface [R(1)] or into calcite [R(4)], hence we cannot generally expect guided modes with real-valued $({k}_{x},{k}_{y})$, even in the limit of negligible material dissipation. When searching for polariton modes at the air–calcite interface in this regime, we need to look for complex-valued wavenumbers satisfying Eq. (1) in the form $q={q}_{{{{{{\rm{r}}}}}}}+{{{{{\rm{i}}}}}}{q}_{{{{{{\rm{i}}}}}}}$, for each real-valued polar angle $\phi$ in the *k*_*x*_*–k*_*y*_ plane (see Supplementary Information Section IV for the reason why $\phi$ needs to be real-valued, a good approximation even when material loss is considered). As a result, Eq. (1) becomes complex-valued, and the choice of appropriate branch cuts and the sign of complex wave vectors become crucial to identify physical solutions^35^ (see Supplementary Information Section I). The resulting IFCs at $\omega=887{{{{{{\rm{cm}}}}}}}^{-1}$ are shown as red lines in Fig. 1g for different values of $\theta$. Interestingly, we observe the emergence of LPs, supported in R(1), R(4) of the in-plane *k*-space, with a lenticular dispersion featuring a closed topology, consistent with the numerical prediction in Fig. 1e. As we increase the angle $\theta$ between the OA and the interface, the extraordinary wave hyperboloids tilt, and hence their projections in the *k*_*x*_*–k*_*y*_ plane move towards the center, forming R(5) and pushing the LP dispersion towards the origin in the ${k}_{x}$ direction, until the critical angle ${\theta }_{{{{{{\rm{c}}}}}}}$ at which the projected hyperbolic contours enter the FSLC, R(4) is split into two isolated regions separated by hyperbolic contours R(5), and hence LPs cannot be supported any longer.

The complex-valued solutions of Eq. (1) shown in Fig. 1e, g constitute the LP spectrum, which effectively hybridizes bulk ordinary waves in calcite with confined extraordinary surface modes, leading to a new form of polariton. Its hybrid features strongly depend on the in-plane momentum: for (*k*_*x*_*, k*_*y*_) parallel or adjacent to the direction of the OA, LPs display surface polariton ($\theta={0}^{^\circ }$) or g-HP like $(\theta\, \ne \,{0}^{^\circ })$ features, with power flow nearly parallel to the interface and smaller radiation leakage, implying four lobes of directional emission into the bulk. When the in-plane wave vector rotates away from the OA, the LPs are more strongly coupled to the bulk ordinary wave with skewed wavefronts. Hence their radiation leakage is larger and ${q}_{{{{{{\rm{i}}}}}}}$ increases outside the FSLC (see Supplementary Information Sections I and III for an extended discussion of the azimuthal dependence of LPs and their eigenmodal field and Poynting vector distributions). The high-symmetry points *k*_*y*_ = 0 (*ϕ* = 0°) and *k*_*x*_ = 0 (*ϕ* = 90°) are particularly interesting, as they correspond to the two extremes of the LPs hybrid nature: at *k*_*y*_ = 0, the LP is purely bound to the interface, while at *k*_*x*_ = 0 it is purely refractive and coupled to the ordinary bulk mode, corresponding to Brewster mode with p-polarization (see Supplementary Information Section II). Notably at these two extremes, the imaginary part of the wavevector *q*_*i*_ vanishes. LPs can emerge also in the transparent regime, for example around *ω* = 890 cm^−1^. In this scenario, the IFCs follow a similar lenticular dispersion, but they are discontinuous when the direction of the wave vector comes close to the OA, because their dispersion intersects the extraordinary wave light line, which is now elliptical in shape (see Supplementary Information Fig. 6).

### Directionality of LPs

**Fig. 2:**
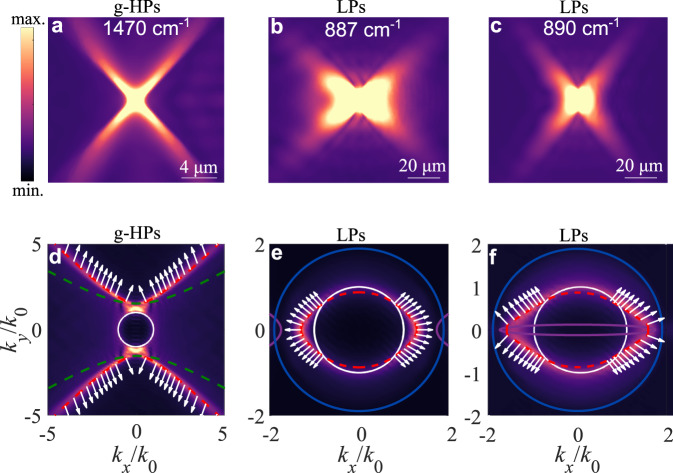
Directional propagation of LPs. **a**–**c** Electric field amplitudes of polaritons in real space showing directional propagation at frequency: **a** $\omega=1470\,{{{{{{\rm{cm}}}}}}}^{-1}$, corresponding to g-HPs in the type-II hyperbolic regime; **b** $\omega=887\,{{{{{{\rm{cm}}}}}}}^{-1}$, corresponding to LPs in type-I hyperbolic regime; **c** $\omega=890\,{{{{{{\rm{cm}}}}}}}^{-1}$, corresponding to LPs in the transparent regime. In all cases $\theta={23.3}^{^\circ }$. **d**–**f** Corresponding in-plane dispersion (red curves) overlaid with Fourier spectra of the ${{{{{{\rm{E}}}}}}}_{z}$ fields from numerical simulations and normalized Poynting vectors (white arrows) of: **d** g-HPs at frequency $\omega=1470\,{{{{{{\rm{cm}}}}}}}^{-1}$ calculated above the interface (in the air); **e** LPs at frequency $\omega=887\,{{{{{{\rm{cm}}}}}}}^{-1}$; **f**. LPs at frequency $\omega=890\,{{{{{{\rm{cm}}}}}}}^{-1}$. All **d**–**f** are calculated from Eq. (1). All lines in **d**–**f** correspond to the same quantities as lines in Fig. 1.

As shown in Fig. 1a(right panel), d, LPs support peculiar directional features in the interface plane, despite being associated with closed contours. The lenticular contours are directly responsible for the directional in-plane propagation of LPs, providing a much stronger overall directionality than elliptical or circular dispersion contours. In order to explain this unexpected property of the lenticular dispersion, we analyze fields and Poynting vectors of LPs on the air-calcite interface plane for $\theta={23.3}^{^\circ }$ in Fig. 2. In particular, we compare the magnitude of the electric field distribution of g-HPs (Fig. 2a) and LPs (Fig. 2b, c) in real space, and overlay the FT from their ${{{{{{\rm{E}}}}}}}_{z}$ fields with their IFCs obtained from Eq. (1) (Fig. 2d–f), considering realistic calcite loss. The emission directionality is associated with the Poynting vector direction for all in-plane momenta, which is normal or nearly normal to the IFCs for surface polaritons. As shown in Fig. 2d, the Poynting vectors of g-HPs for each hyperbolic branch are mostly aligned to each other, due to the open topology of their dispersion, leading to highly directional propagation in real space. LPs, due to their peculiar lenticular dispersion, also feature power flows that are uncommonly parallel to each other in the same quadrant, exhibiting directional propagation in real space (Fig. 2b, c). This is surprising since their IFC as shown in Fig. 2e, f has a closed or nearly closed topology for $\omega=887\,{{{{{{\rm{cm}}}}}}}^{-1}$ and $\omega=890\,{{{{{{\rm{cm}}}}}}}^{-1}$, corresponding to the type-I hyperbolic and transparent regimes, respectively. The brighter spectra outside the FSLC of the FT in Fig. 2e, f indicate the power flow in the plane is mainly associated with polaritons outside the FSLC, which indeed have Poynting vectors (white color in Fig. 2e, f) well aligned across the dispersion curve, far more than they would be with a conventional circular or elliptic dispersion. This directionality is fostered by the fact that the LP propagation length is almost constant along the IFC outside the FSLC, despite the increasing radiation leakage for increasing $\left|\phi \right|$. This effect arises because the effective material loss of the hybridized mode simultaneously decreases for increasing $\left|\phi \right|$, leading to enhanced directionality (see Supplementary Information Section III for an extended discussion and Section VII for a quantitative study of directionality).

### Polariton spectroscopy and far-field reflectance spectroscopy

**Fig. 3:**
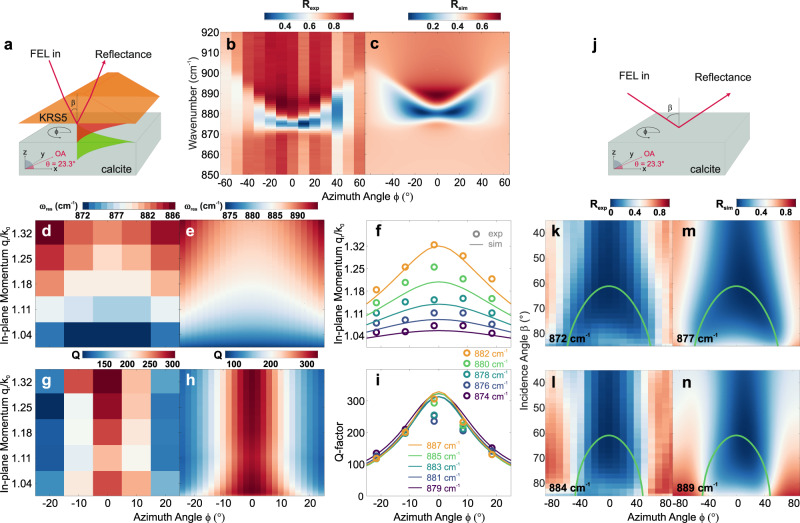
Otto-type prism coupling measurement for the experimental observation of LPs. **a** Otto-type prism coupling configuration. **b** Azimuthal dispersion extracted from experimental data and **c** transfer matrix simulations for calcite (100), with the optical axis tilted by $\theta={23.3}^{^\circ }$ with respect to the interface (as displayed in **a**). These simulations were performed by taking into account the presence of a Thallium Bromoiodide (KRS5) prism when setting up the material system and calculating for each configuration the corresponding reflectance spectrum. The in-plane momentum is fixed at $\frac{{q}_{{{{{{\rm{r}}}}}}}}{{k}_{0}}=1.07$. **d** experimental and **e** simulated polariton resonance frequency map. The polariton resonance frequencies in the simulated maps are derived from the imaginary part of the reflection coefficients for *p*-polarized light ${\mathfrak{I}}({r}_{{{{{{\rm{pp}}}}}}})$. **f** Simulated (lines) and experimental (circles) IFCs at multiple frequencies, demonstratin**g** the lenticular dispersion of LPs. **g** Experimental and **h** simulated LP Q-factors as a function of in-plane momentum. **i** Simulated (lines) and experimental (circles) LP Q-factors along the IFCs at multiple frequencies. **j** Experimental geometry for LP dispersion measurement inside the FSLC. **k**, **l** Experimental isofrequency reflectance maps and **m**, **n** respective simulations for (100) calcite. The green solid lines show the analytical LP dispersion using Eq. (1). Note that we observe a spectral offset of ~$5\,{{{{{{\rm{cm}}}}}}}^{-1}$ between experiment and simulations consistently for all measurements.

To experimentally observe LPs and map their peculiar dispersion in and out of the FSLC, we performed both polariton spectroscopy and far-field reflectance spectroscopy. First, we probed the LP dispersion in the frequency domain using Otto-type prism coupling^32,38^ for an air–calcite (100) interface with the OA slanted by $\theta=23.3^\circ$ (see Methods for details). We placed a prism made of dielectric Thallium Bromoiodide (KRS5) above the air-calcite interface with a fixed air gap ${d}_{{{{{{\rm{gap}}}}}}}=4\,{{{{{\rm{\mu }}}}}}{{{{{\rm{m}}}}}}$, and acquired reflectance spectra at the prism back-surface for several azimuthal angles $\phi$ between the incidence plane and the OA, see Fig. 3a. By fixing the incidence angle $\beta$, we select a specific value of in-plane momentum $\frac{{q}_{{{{{{\rm{r}}}}}}}}{{k}_{0}}$ of the excited evanescent waves. The resulting reflectance spectra for $\beta=27^\circ$, corresponding to $\frac{{q}_{{{{{{\rm{r}}}}}}}}{{k}_{0}}=1.075$ at $\omega=887$ cm^−1^, are shown in Fig. 3b. We observe a high-quality polariton resonance at $\phi=0^\circ$ (OA parallel to the incidence plane), which blue-shifts and quickly broadens as we rotate the sample in either direction about its surface normal. The experimental data are in good agreement with transfer matrix simulations (see Methods) shown in Fig. 3c. In order to map the in-plane dispersion of LPs, we acquired the azimuthal dependence for several incidence angles (see Supplementary Information section V for the full data set), and extracted the resonance positions, as well as their quality (*Q*)-factors. The retrieved resonance frequency map shown in Fig. 3d, in excellent agreement with the simulated data in Fig. 3e, displays the peculiar lenticular LP dispersion. This is confirmed in the retrieved IFCs from these data (see Methods), shown in Fig. 3f, which provide direct experimental proof of the lenticular in-plane dispersion of LPs. Further experiments (shown in Supplementary Information, Figs. S9 and S10) confirm similar features for $\theta=48.5^\circ$ using calcite and for $\theta=0^\circ$ using α-Quartz.

**Fig. 4:**
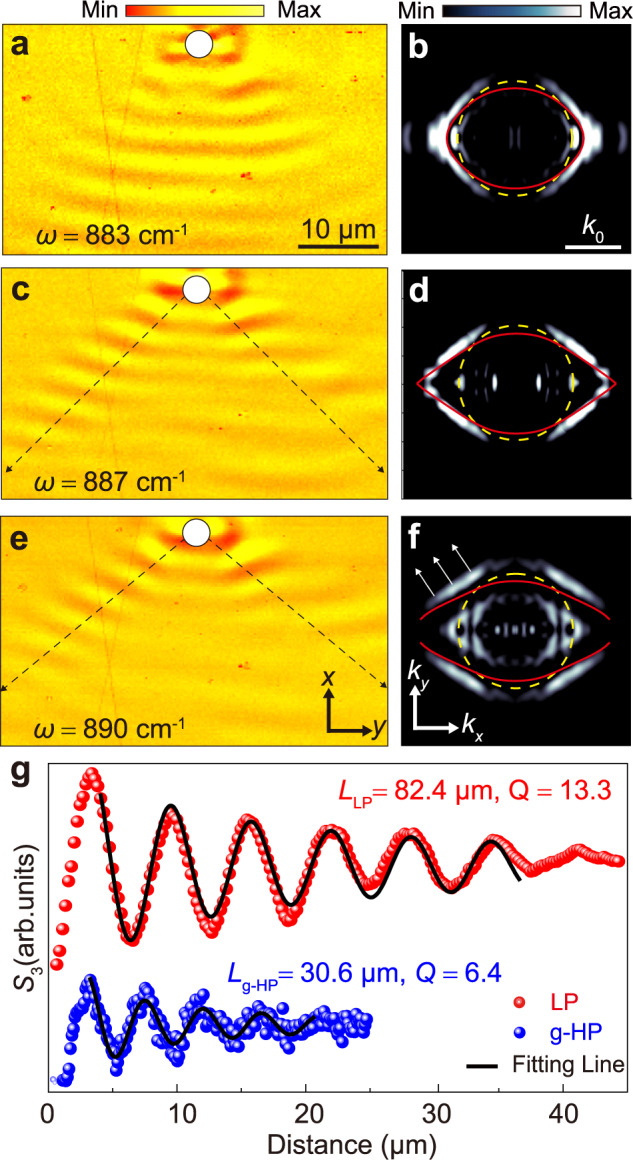
Near-field imaging of directional propagation of LPs in real space. **a**, **c**, **e** Nanoscale imaging of LPs at *ω* = 883 cm^−1^, 887 cm^−1^, 890 cm^−1^, generated by recording the amplitude of the measured signal, including both type-I hyperbolic and transparent regimes. The indicated directions in **c** and **e** are closely aligned with the confined directional fields, where the black arrows are estimated from simulations. **b**, **d**, **f** Corrected FT (see Supplementary Information Fig. S11 for details) of near-field distribution in (**a**, **c**, **e**) (Note that the dispersions are flipped by 90° clockwise here for clearer presentation). The dispersions are corrected by considering the shift in transverse wave vector *k*_*x*_ caused by oblique incidence; see Supplementary Information for details. The dashed yellow lines indicate the FSLC, theory dispersions of LPs are marked by solid red lines. The directive LPs in (**e**) are expected in the flattened iso-frequency dispersion contour marked white arrows in (**f**). **g** The comparison of LPs and g-HPs damping extracted from experiments. The red balls show the s-SNOM optical signal(*S*_3_) of LPs from (**e**), and the blue balls show the signal of g-HPs at *ω* = 1460 cm^−1^ (shown in Fig. S17), which are both normalized on a smooth background signal. The black solid line indicates the fitting results by a modified damped wave function: *S*_3_ = *x*^−0.5^ · *e*^*-γx*^*·* sin*(kx)*, where *x* is the distance away from the source, the relative propagation length (*Q*) can be estimated from *k*/*γ*, *L*_*p*_ = 1/*γ*.

The experimental and simulated Q-factors of LPs in calcite are shown in Fig. 3g, h, respectively. We retrieved Q-factor values along the IFCs at multiple frequencies, as shown in Fig. 3i. Remarkably, we are generally able to observe very large Q-factors, exceeding 300 at $\phi=0^\circ$ and *Q* ~ 150 at $\left|\phi \right |=20^\circ$, demonstrating a long lifetime for LPs, despite their leaky nature, a consequence of the low damping of the associated optical phonon polarized in the OA direction^39^, leading to unprecedented directional radiation leakage. Strikingly, the Q-factors rapidly decrease for increasing $\left| \phi \right|,$ for all momenta (Fig. 3h) and at all frequencies (Fig. 3i). Hence, the LPs experience a steep increase in optical loss as the polarization plane is rotated away from the OA. Nonetheless, low-loss LP propagation also in directions away from the OA is well-supported since the decreasing Q-factor is compensated by an increasing group velocity (see Supplementary Information IX). Thus, the azimuthal dependence of the LP Q-factor is indeed consistent with the real-space propagation patterns predicted in Fig. 2b, c, where off-OA directions dominate the propagation patterns, as experimentally verified in Fig. 4.

Before discussing these near-field measurements, we additionally performed far-field experiments to verify the LP dispersion also inside the FSLC. For this purpose, we acquired free-space reflectance spectra of calcite (100) for a series of large incidence angles $\beta$ and azimuthal angles $\phi$, see Fig. 3j for the experimental arrangement. The reflectance data allows for extraction of isofrequency reflectance maps, as shown in Fig. 3k, l (see Supplementary Information X for the full data set), in excellent agreement with the simulations shown in Fig. 3m, n. In these data, the reflectance minima mark the LP, which here—inside the FSLC—can be understood as a Brewster-like mode with strong azimuthal dispersion. It is this component of the LPs that allows free-space coupling and directional thermal emission, absent in any other polariton platform.

### Near-field optical microscopy

In order to visualize the directional excitation and long-range propagation of LPs in real space, we performed near-field imaging of LPs on an air–calcite interface with $\theta=23.3^\circ$. A gold nanodisk (diameter $D$ = 4 μm) was deposited on the interface as an optical antenna that concentrates *p*-polarized mid-infrared illumination (with an oblique incident angle $\beta$ = 30° along *z*) into a localized hotspot, thus enabling the launching of LPs (as shown in Fig. 4a and Supplementary Information Fig. S11). The launched field *E*_p_ propagates along the surface, interfering with the incident field *E*_in_, and the interference field is mapped by a sharp metallic tip of scattering-type scanning near-field optical microscope (s-SNOM) with nanoscale resolution. Figure 4a presents the near-field measured amplitude of disk-launched LPs at $\omega=883\,{{{{{{\rm{cm}}}}}}}^{-1}$, revealing LPs with an arc-type wavefront. The interference between LPs and the incident field renders the wavenumber of LPs slightly enlarged in ${k}_{x}$ by the interference factor $\Delta={k}_{0}*\sin \beta$^31^. The desired signal-to-noise ratio is calculated (Supplementary Information Fig. S16) to estimate the quality of the experimental results. Figure 4b presents the FT of the near-field distribution in Fig. 4a, confirming the peculiar lenticular shape of IFC and its closed topology in *k*-space (details in Supplementary Information Figs. S11 and S12). The yellow dashed line indicates the FSLC and the red solid line presents the theoretical dispersion, exhibiting good agreement between theoretical and experimental results. At frequency $\omega=887\,{{{{{{\rm{cm}}}}}}}^{-1}$ the measured LP fields exhibit higher directionality, consistent with the more pronounced flattening of the IFC shown in Fig. 4d, again agreeing well with our theoretical predictions. The directional propagation of LPs observed here is consistent with our numerical simulations at oblique illumination (Supplementary Information S13). Figure 4e, f shows (e) the spatial field distribution of LPs and (f) the corresponding IFC at $\omega=890\,{{{{{{\rm{cm}}}}}}}^{-1}$ in the transparent regime, in which the directionality of propagation is further enhanced, now being strongly dominated by the flat parts of the lenticular IFC. Both arrows estimated from simulation in Fig. 4c, e are closely aligned with the confined directional fields, demonstrating how the direction of polariton propagation can be fine-tuned by detuning the illumination frequency. More results from additional measurements and comparisons between the theoretical and experimental results can be found in Supplementary Information Figs. S14 and S15. Directional propagation of LPs is also sustained by tuning the OA orientation of calcite (Supplementary Information Fig. S16), consistent with our theoretical prediction. The real space imaging of LPs and recorded dispersion further validate the observation of a new form of polaritons, directional and leaky in nature and significantly distinct from hyperbolic surface and volume polaritons. To quantify the propagation damping of LPs, we extract the lines of the near-field signal along the left dashed line in Fig. 4e; the decay of the fringes away from the Au antenna can be attributed to the damping of LPs and geometrical spreading^40^. The relative propagation length of the measured LPs is estimated to be 13.3, which is larger than the estimated value of 6.4 for g-HPs, thus indicating a lower propagation loss for LPs. The detailed comparison between LPs and g-HPs can be found in Supplementary Information Fig. S17.

## Discussion

In this work, we have unveiled with theoretical investigations, *k*-space, and real-space near-field measurements, a new form of polariton, living at the interface of highly anisotropic materials, leaky in nature and with unique spectral features stemming from the hybridization between refractive bulk modes and surface-bound polaritons. We experimentally demonstrated its exotic dispersion features in and out of FSLC and directional propagation properties, probing via an Otto-type prism coupling geometry and in real space via near-field imaging. The observed LPs feature both lenticular dispersion and associated in-plane directionality, enabling us to bridge the concepts of leaky waves and anisotropic surface polaritons. Distinct from conventional leaky wave phenomena occurring in structured interfaces with subwavelength, carefully tailored geometries or isotropic material, our work demonstrates the existence of these hybrid modes in natural, unstructured, and anisotropic crystals.

We emphasize that the fundamental nature of LPs and g-HPs is very different. LPs stem from the hybridization between surface polaritons and propagating bulk modes. Therefore, their in-plane momentum is generally complex, even in the absence of material loss. As a result, LPs possess both tilted wavefronts and Poynting vectors canted away from the interface, whose magnitude can grow as they move away from the interface because of their forward leaky-wave nature. Ghost polaritons, on the contrary, have a complex out-of-plane momentum component, but their in-plane component is real-valued. In fact, g-HPs are surface polaritons that have slanted phase wavefronts, but their Poynting vector is parallel to the interface. Hence, the power flow necessarily decays away from the interface like conventional surface waves. In addition, g-HPs usually emerge in uniaxial media like calcite when the OA is tilted with respect to the interface. LPs, as our complex eigenvalue analysis shows, can exist in a wide range of natural anisotropic materials as long as a leaky mode can hybridize with guided modes at the interface. The formation of LPs can hence be generalized to a wide range of natural and engineered photonic systems, including hyperbolic and zero-index metamaterials^41–43^. Our additional experiments on α-quartz (Supplementary Information Fig. S10) provide direct evidence of LPs in other naturally hyperbolic materials. While their propagation along the interface is sub-diffractive, LPs are complementary to conventional hyperbolic polaritons, with more limited confinement, which may be traded for easier excitation and longer lifetimes with Q-factors exceeding 300 and longer propagation lengths exceeding 13, as we explicitly demonstrate in Fig. 3i and Fig. 4g.

Despite emerging at the surface of an anisotropic medium, LPs display confinement factors that may not be as large as conventional hyperbolic polaritons because of the closer distance of their dispersion to FSLC. However, the hyperbolicity of the medium and associated strong anisotropy enables the peculiar dispersion features, directional in-plane propagation, and, interestingly, directional radiation leakage in the far-field (Fig. S20), a phenomenon not achievable in other platforms. Furthermore, highly confined volume hyperbolic modes have small group velocities, such that it is challenging to leverage those strongly confined modes for energy transport in photonic platforms. In this context, LPs can provide reasonable group velocities, long propagation distances, and high *Q*-factors, offering unprecedented opportunities for nanophotonic integration. Thus, we anticipate that the directional LPs with lenticular dispersion, combined with wavefront engineering, may have important implications to further expand the role of polaritonics in nanoscale applications from mid-infrared to far-infrared imaging, bio-sensing, and nanobeam guiding on integrated platforms.

## Methods

### Materials and fabrication

We use commercially available bulk calcite single crystals (Kejing, a Chinese company) with the characteristic plane (100) and (104), corresponding to the cases of OA with *θ* = 23.3° and 48.5° to surfaces, and an a-cut α-Quartz sample (used for Otto-type prism coupling experiments, Supplementary Information Fig. S10) were purchased from MaTeck GmbH (Germany). For s-SNOM sample preparation, the electron beam resist (PMMA) and then a conductive polymer were spin-coated on the calcite surface. An electron beam lithography system was used to pattern the gold disk antennas. The standard lift-off procedure was put to use after electron-beam evaporation of a Cr/Au (3 nm/40 nm) layer on the developed resist.

### Otto-type prism coupling measurements

An Otto-type prism coupling experiment is employed to optically excite nonradiative surface waves, see Fig. 3a. In the Otto geometry, the sample is brought in close vicinity to the prism. Incident light enters the prism at an angle *β* greater than the critical angle for total internal reflection producing evanescent waves on the back side of the prism that can couple to surface waves on the sample. By measuring the intensity of the reflected light over a certain frequency range of the incident radiation, the polariton resonances are detected as absorption dips in the reflectance spectrum. The Otto geometry enables experimental control over the excitation efficiency via tunability of the air gap width ${d}_{{{{{{\rm{gap}}}}}}}$. In the experiment, the prism-sample spacing was kept fixed at ${d}_{{{{{{\rm{gap}}}}}}}\, \approx \,4{{{{{\rm{\mu }}}}}}{{{{{\rm{m}}}}}}$. The air gap was measured by white-light interferometry, which can determine gap sizes from 500 nm up to the highest resolvable coherence length of 60 µm.

As a light source, we employ a mid-infrared free electron laser (FEL) that provides high power, tunable radiation (3–50 µm) with a narrow bandwidth ($\sim 0.3\%$), further detail on the FHI FEL are reported elsewhere^40^. In particular, we focus on the spectral range between $850\, {{{{{\rm{and}}}}}}\, 920\,{{{{{{\rm{cm}}}}}}}^{-1}$, corresponding to the lower reststrahlen band of calcite, where LPs are supported. The FEL allows us to perform narrow-band spectroscopy, i.e. to scan the frequency, providing the full reflectance spectra, at a selected combination of in-plane momentum $\frac{{q}_{{{{{{\rm{r}}}}}}}}{{k}_{0}}$ and azimuth angle $\phi$. The two parameters $\frac{{q}_{{{{{{\rm{r}}}}}}}}{{k}_{0}}$ and $\phi$ correspond to magnitude and propagation direction of the in-plane momentum of the incident beam, respectively. Different in-plane momenta are experimentally accessed by varying the incidence angle $\beta$ (indicated in Fig. 3a), which is achieved by rotating the entire Otto geometry, whereas changes in the azimuth angle are obtained by rotating the sample.

For the azimuthal dispersion shown in Fig. 3b, the measurements were taken at a fixed incidence angle of $27^\circ$ (corresponding to $\frac{{q}_{{{{{{\rm{r}}}}}}}}{{k}_{0}}=1.075$), whilst the resonance frequency maps depicted in Fig. 3d (and the relative Q-factors in Fig. 3g) were obtained by varying both parameters. In the latter case, the Otto reflectance spectra were taken for five different azimuth angles *ϕ*, at *β* = 26°, 28°, 30°, 32°, and 34° corresponding to in-plane momenta of $\frac{{q}_{{{{{{\rm{r}}}}}}}}{{k}_{0}}\, \approx \,1.04,\,1.11,\,1.18,\,1.25,$ and $1.32$ (at about $900\,{{{{{{\rm{cm}}}}}}}^{-1}$). The reflectance minima (representing the polariton resonances) and their Q-factors were extracted, as displayed in Fig. 3d, g, respectively. These maps enabled us to additionally calculate the in-plane dispersion curves (IFCs) at multiple frequencies (Fig. 3f) by interpolating the momentum for a given frequency in the frequency–momentum dispersion for each measured azimuth angle. The corresponding Q-factors (plotted in Fig. 3i) were derived by interpolating the FWHM of each reflectance dip for the in-plane momenta obtained from the previous interpolation in the FWHM-momentum dependence. To define the optical axis, the absolute azimuth offset $\triangle \phi$ of the sample was computed by minimizing the sum of squared deviations of the experimentally derived IFCs (circles in Fig. 3f) from the simulated curves (lines in Fig. 3f), resulting in a rotation of the optical axis with respect to the *x*-axis of the laboratory coordinate system of $\Delta \phi=-1.6^\circ$. The experimental data (circles) in Fig. 3f, i have been shifted accordingly.

### Far-field reflectance measurements

A home-built double goniometer is employed for *β*–2*β* scanning of the incidence angle by rotating the sample by *β* and the reflectance detector by 2*β*, respectively. The FEL beam is mildly focused (spot size ~500 µm) onto the sample under grazing incidence. The reflected intensity is detected by a pyroelectric detector, whose signal is divided by that of an identical reference detector. Two-dimensional scans of the FEL wavenumber and incidence angle, respectively, are acquired for each sample azimuthal angle. Spectral drift of the FEL is accounted for by online monitoring of the single-shot FEL spectrum and interpolation of the data onto a wavenumber-incidence angle grid during post-processing. Notably, absolute referencing of the reflected intensity is difficult in this arrangement due to finite sample size (resulting in beam clipping at large incidence angles approaching 90°) and sample wobble. To minimize these effects on the measured intensities, we optimized the beam alignment for each azimuthal angle, thereby rendering an absolute reflectance referencing using a reference reflector unreliable. It is, in practice, also impossible to achieve identical absolute intensities despite alignment optimization. We thus normalized the 2D wavenumber-incidence dataset for each azimuth according to the predicted global maximum from the equivalent simulated dataset.

### s-SNOM measurements

For near-field imaging, a commercial s-SNOM system (Neaspec GmbH, Munich, Germany) based on an atomic force microscope (AFM) was used. A metalized AFM tip (silicon coated with platinum) oscillates vertically with an amplitude of about 70 nm at a frequency of *f* ≈ 270 kHz and is illuminated by a p-polarized mid-infrared quantum cascade laser with operation frequency ranging from 870 to 950 cm^−1^. The signal-to-noise ratio of the SNOM measurement is relatively low as the illuminated frequency approaches the lower frequency limit of the laser. The sharp apex of the tip offers the nanoscale resolution for recording LPs. The scattered signals are collected with a pseudo-heterodyne interferometer for suppressing background signals. All near-field signals are demodulated at a higher harmonic *nΩ* (*n* ≥ 3), yielding both amplitude *s*_n_ and phase *φ*_*n*_ images. Figure 4 shows amplitude *s*_3_ images of LPs.

### Transfer matrix technique

The calculations of all simulated data shown in Fig. 3 were performed using a $4\times 4$ transfer matrix formalism^44^. The formalism allows for the calculation of reflection and transmission coefficients in any number of stratified media with arbitrary dielectric tensor, which allows to account for the anisotropy of our samples.

### Numerical simulations

COMSOL version 5.6 was used for simulating point dipole excitation of both g-HPs and LPs at the interface of calcite and air. A point dipole was placed 200 nm above the surface of a semi-spherical calcite, where spherical scattering boundary conditions were used on the spherical boundaries to absorb all outgoing radiation. The radius of semi-spherical calcite was sufficiently large $R=80$ µm such that the wave is sufficiently damped when it reaches the boundary and thus has little impact on the results. The dielectric function and parameters of calcite were adopted from ref. ^31^.

## Supplementary information

Supplementary Materials

Peer review file

## Data Availability

The prism coupling and far-field reflectance experimental data and analysis scripts can be accessed at 10.5281/zenodo.7804555. All other data featured in the figures of this paper and other findings of this study are available from the corresponding author upon reasonable request.

## Code availability

The codes that support the findings of this study are available from the corresponding authors upon reasonable request.

## References

[1] Basov DN, Fogler MM, de Abajo FJG (2016). Polaritons in van der Waals materials. Science.

[2] Low T (2017). Polaritons in layered two-dimensional materials. Nat. Mater..

[3] Foteinopoulou S, Devarapu GCR, Subramania GS, Krishna S, Wasserman D (2019). Phonon-polaritonics: enabling powerful capabilities for infrared photonics. Nanophotonics.

[4] Hu GW, Shen JL, Qiu CW, Alu A, Dai SY (2020). Phonon polaritons and hyperbolic response in van der Waals Materials. Adv. Opt. Mater..

[5] Zhang Q (2021). Interface nano-optics with van der Waals polaritons. Nature.

[6] Sun JB, Litchinitser NM, Zhou J (2014). Indefinite by nature: from ultraviolet to terahertz. ACS Photonics.

[7] Esslinger M (2014). Tetradymites as natural hyperbolic materials for the near-infrared to visible. ACS Photonics.

[8] Dai S (2014). Tunable phonon polaritons in atomically thin van der Waals crystals of boron nitride. Science.

[9] Ma WL (2018). In-plane anisotropic and ultra-low-loss polaritons in a natural van der Waals crystal. Nature.

[10] Taboada-Gutierrez J (2020). Broad spectral tuning of ultra-low-loss polaritons in a van der Waals crystal by intercalation. Nat. Mater..

[11] Brar VW (2014). Hybrid surface-phonon-plasmon polariton modes in graphene/monolayer h-BN heterostructures. Nano Lett..

[12] Dai S (2015). Graphene on hexagonal boron nitride as a tunable hyperbolic metamaterial. Nat. Nanotechnol..

[13] Caldwell JD (2014). Sub-diffractional volume-confined polaritons in the natural hyperbolic material hexagonal boron nitride. Nat. Commun..

[14] Dai S (2015). Subdiffractional focusing and guiding of polaritonic rays in a natural hyperbolic material. Nat. Commun..

[15] Li PN (2015). Hyperbolic phonon-polaritons in boron nitride for near-field optical imaging and focusing. Nat. Commun..

[16] Tomadin A, Principi A, Song JCW, Levitov LS, Polini M (2015). Accessing phonon polaritons in hyperbolic crystals by angle-resolved photoemission spectroscopy. Phys. Rev. Lett..

[17] Kumar A, Low T, Fung KH, Avouris P, Fang NX (2015). Tunable light-matter interaction and the role of hyperbolicity in graphene-hBN system. Nano Lett..

[18] Giles AJ (2018). Ultralow-loss polaritons in isotopically pure boron nitride. Nat. Mater..

[19] Dai SY (2018). Manipulation and steering of hyperbolic surface polaritons in hexagonal boron nitride. Adv. Mater..

[20] Zheng ZB (2019). A mid-infrared biaxial hyperbolic van der Waals crystal. Sci. Adv..

[21] Duan J (2021). Enabling propagation of anisotropic polaritons along forbidden directions via a topological transition. Sci. Adv..

[22] Zeng Y (2022). Tailoring Topological Transitions of Anisotropic Polaritons by Interface Engineering in Biaxial Crystals. Nano Lett..

[23] Sun FS (2021). Polariton waveguide modes in two-dimensional van der Waals crystals: an analytical model and correlative nano-imaging. Nanoscale.

[24] Álvarez-Pérez, G., Voronin, K. V., Volkov, V. S., Alonso-González, P. & Nikitin, A. Y. Analytical approximations for the dispersion of electromagnetic modes in slabs of biaxial crystals. *Phy. Rev. B* **100**, 235408 (2019).

[25] Hu GW (2020). Topological polaritons and photonic magic angles in twisted alpha-MoO(3)bilayers. Nature.

[26] Chen MY (2020). Configurable phonon polaritons in twisted alpha-MoO3. Nat. Mater..

[27] Duan J (2020). Twisted Nano-Optics: Manipulating Light at the Nanoscale with Twisted Phonon Polaritonic Slabs. Nano Lett..

[28] Zheng Z (2020). Phonon Polaritons in Twisted Double-Layers of Hyperbolic van der Waals Crystals. Nano Lett..

[29] Frech R, Nichols H (1978). Infrared reflectivity of calcite—oblique phonons. Phys. Rev. B.

[30] Breslin VM, Ratchford DC, Giles AJ, Dunkelberger AD, Owrutsky JC (2021). Hyperbolic phonon polariton resonances in calcite nanopillars. Opt. Express.

[31] Ma WL (2021). Ghost hyperbolic surface polaritons in bulk anisotropic crystals. Nature.

[32] Passler N (2022). Hyperbolic shear polaritons in low-symmetry crystals. Nature.

[33] Takayama O (2008). Dyakonov surface waves: a review. Electromagnetics.

[34] Marcuvitz N (1956). On field representations in terms of leaky modes or eigenmodes. IRE Trans. Antennas Propag..

[35] Lampariello P, Frezza F, Oliner AA (1990). The transition region between bound-wave and leaky-wave ranges for a partially dielectric-loaded open guiding structure. IEEE Trans. Microw. Theory.

[36] Jackson DR, Caloz C, Itoh T (2012). Leaky-wave antennas. Proc. IEEE.

[37] Monticone F, Alu A (2015). Leaky-wave theory, techniques, and applications: from microwaves to visible frequencies. Proc. IEEE.

[38] Passler NC (2017). Second-harmonic generation from critically coupled surface phonon polaritons. ACS Photonics.

[39] Hellwege KH, Lesch W, Plihal M, Schaack G (1970). Zwei-phononen-absorptionsspektren und dispersion der schwingungszweige in kristallen der kalkspatstruktur. Z. Phys. A Hadrons Nucl..

[40] Li P (2018). Infrared hyperbolic metasurface based on nanostructured van der Waals materials. Science.

[41] Poddubny A, Iorsh I, Belov P, Kivshar Y (2013). Hyperbolic metamaterials. Nat. Photonics.

[42] Huo PC, Zhang S, Liang YZ, Lu YQ, Xu T (2019). Hyperbolic metamaterials and metasurfaces: fundamentals and applications. Adv. Opt. Mater..

[43] Shen L (2020). Broadband enhancement of on-chip single-photon extraction via tilted hyperbolic metamaterials. Appl. Phys. Rev..

[44] Passler NC, Paarmann A (2019). Generalized 4 × 4 matrix formalism for light propagation in anisotropic stratified media: study of surface phonon polaritons in polar dielectric heterostructures: erratum. J. Opt. Soc. Am. B.

