# Supplementary material for: Observation of directional leaky polaritons at anisotropic crystal interfaces
Source: Nat Commun. 2023 May 18;14:2845. doi: 10.1038/s41467-023-38326-7 (PMC10195857; doi:10.1038/s41467-023-38326-7)
Supplement: Supplementary file 1 — Supplementary Materials [file 41467_2023_38326_MOESM1_ESM.pdf]

Supplementary Information for

"Observation of Directional Leaky Polaritons over Anisotropic Crystal Interfaces"

Xiang Ni,[1,6,7] Giulia Carini,[2,7] Weiliang Ma[3,7], Enrico Maria Renzi,[1] Emanuele Galiffi,[1] Sören Wasserroth,[2] Martin Wolf,[2] Peining Li[3,4*], Alexander Paarmann,[2,*] Andrea Alù,[1,5*]

[1]Photonics Initiative, Advanced Science Research Center, City University of New York, New York, NY 10031, USA

[2] Fritz Haber Institute of the Max Planck Society, Berlin, Germany

[3]School of Optical and Electronic Information, Wuhan National Laboratory for Optoelectronics and Wuhan National high Magnetic Field Center, Huazhong University of Science and Technology, Wuhan, China

[4]Optics Valley Laboratory, Hubei 430074, China

[5]Physics Program, Graduate Center, City University of New York, New York, NY 10016, USA

[6]School of Physics and Electronics, Central South University, Changsha, Hunan, 410083, China.

[7]These authors contributed equally

[*]Corresponding authors: lipn@hust.edu.cn, alexander.paarmann@fhi-berlin.mpg.de, aalu@gc.cuny.edu

Table of Contents

I. Complex eigenmode study for leaky polaritons 3

a. Bulk eigenmodes in calcite 3

b. Complex secular equation for leaky polaritons in lossless system 6

c. Branch cut and $\langle Sz \rangle$ diagram 7

d. Field distributions and Poynting vectors 9

II. Solutions of leaky polaritons at symmetry points 15

a. $\mathrm{ky} = 0$ 15

b. $\mathrm{kx} = 0$ 16

III. Directional hybridization and in-plane damping rate 16

IV. Explanation for the parallel condition of qi and qr 18

V. Experimental data from Otto-type polariton spectroscopy measurement 19

a. Data for calcite with $\theta = 23.3°$ 19

b. Data for calcite with $\theta = 48.5°$ 19

c. Data for SiO_2 21
VI. Near field imaging experiment 22
a. Experiment for calcite with $\theta = 23.3°$ 22
b. Experiment for calcite with $\theta = 48.5°$ 28
VII. Directionality comparison of in-plane propagation 30
VIII. Directionality comparison of far-field emission 31
IX. Polariton life time and propagation losses 32
X. Experimental data for far-field probing 33
Reference 33

I. Complex eigenmode study for leaky polaritons

a. Bulk eigenmodes in calcite

Here, we study the leaky polariton propagation at the interface between air and a uniaxial material with optical axis (OA) forming a slanted angle θ with respect to its surface (shown in Fig. 1a). We consider in particular calcite, and use the Lorentz-Drude model to fit its permittivity components, $\epsilon_{\parallel}$ and $\epsilon_{\perp}$, as a function of frequency, which are the principal permittivity components parallel and perpendicular to the OA, respectively, as shown in Fig. S1. Two Reststrahlen bands[2], characterized by high optical reflectivity and strong absorption, exist in the spectral range $800 - 1600\mathrm{cm}^{-1}$, which lie between the transverse phonon resonance $\omega_{\mathrm{TO,2}}$, and the longitudinal phonon resonance $\omega_{\mathrm{LO,2}}$, referred to as upper Reststrahlen band (light green region), and between the transverse phonon resonance $\omega_{\mathrm{TO,3}}$, and longitudinal phonon resonance $\omega_{\mathrm{LO,3}}$, as lower Reststrahlen band (light orange and blue region), respectively. The lower Reststrahlen band and its neighborhood are further divided into two regimes: when $\epsilon_{\perp} > 0$ and $\epsilon_{\parallel} < 0$, the bulk dispersion of calcite is in the type-I hyperbolic regime (light orange region); when $\epsilon_{\perp} > 0$ and $0 < \epsilon_{\parallel} < \epsilon_0$, where ϵ_0 is the permittivity in vacuum, it is in the near-zero-index transparent regime (light blue region).

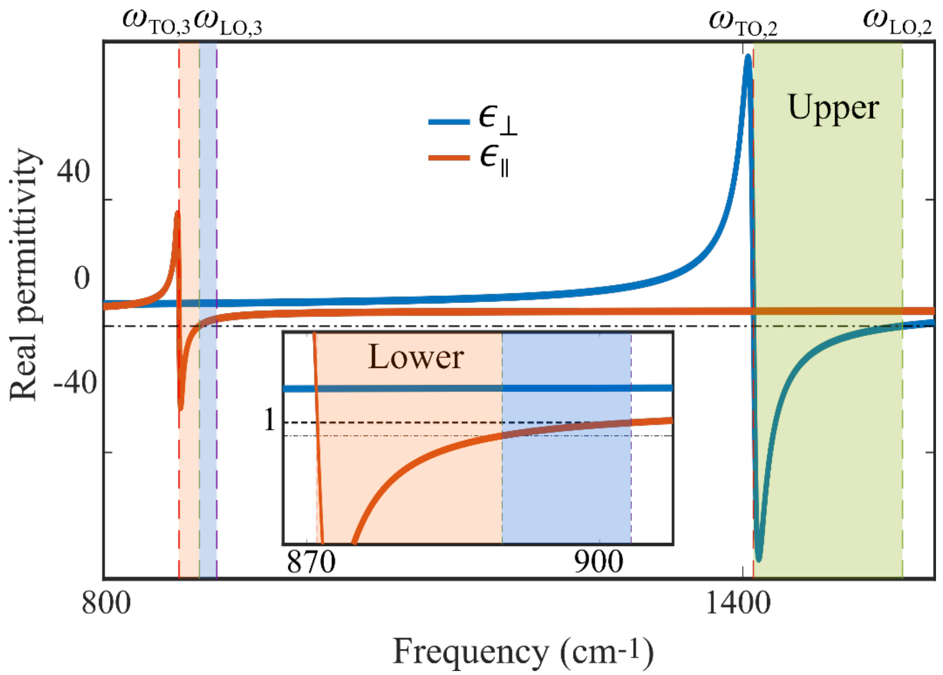

Figure S1. Real permittivity components $\epsilon_{\parallel}$ and $\epsilon_{\perp}$ of calcite crystal. The parameters of the Lorentz-Drude model are taken from Ref. [1].

To understand the nature of leaky polaritons (LPs) studied in our work and obtain their iso-frequency contours, we first study the bulk modes in calcite located in the half-space $z < 0$. The corresponding permittivity tensor in the reference frame aligned with the interface is

$$\hat{\epsilon}_{rot} = \hat{R}_{xz}(\theta)\hat{\epsilon}\hat{R}_{xz}(\theta)^{-1}, \tag{1}$$

where $\hat{\epsilon} = \mathrm{diag}(\epsilon_{\parallel}, \epsilon_{\perp}, \epsilon_{\perp})$, and $\hat{R}_{xz}(\theta) = \begin{pmatrix} \cos(\theta) & 0 & \sin(\theta) \\ 0 & 1 & 0 \\ -\sin(\theta) & 0 & \cos(\theta) \end{pmatrix}$ is a rotation matrix that transforms the permittivity tensor to the reference frame of the interface, where the *z*-axis is parallel to the normal of the interface and the OA lies in the *x-z* plane, as shown by the schematic in Fig. 1a. We assume they allow plane wave solutions $\mathrm{e}^{\mathrm{i}(\boldsymbol{kr}-\omega t)}$, thus the source-free Maxwell equations read:

$$\begin{pmatrix} \epsilon_{xx}k_0^2 - (k_z^2 + k_y^2) & k_x k_y & \epsilon_{xz}k_0^2 + k_x k_z \\ k_x k_y & \epsilon_{yy}k_0^2 - (k_z^2 + k_x^2) & k_y k_z \\ \epsilon_{xz}k_0^2 + k_x k_z & k_y k_z & \epsilon_{zz}k_0^2 - (k_y^2 + k_x^2) \end{pmatrix} \begin{pmatrix} E_x \\ E_y \\ E_z \end{pmatrix} = 0, \quad (2)$$

Where

$$\epsilon_{xx} = \frac{1}{2}(\epsilon_{\perp} + \epsilon_{\parallel} - (\epsilon_{\perp} - \epsilon_{\parallel})\cos(2\theta)),$$

$$\epsilon_{yy} = \epsilon_{\perp}, \epsilon_{zz} = \frac{1}{2}(\epsilon_{\perp} + \epsilon_{\parallel} + (\epsilon_{\perp} - \epsilon_{\parallel})\cos(2\theta)),$$

$$\epsilon_{xz} = (\epsilon_{\perp} - \epsilon_{\parallel})\cos(\theta)\sin(\theta),$$

and $k_0 = \frac{\omega}{c}$. Next we apply a new rotation, this time in the *x-y* plane: $\hat{R}_{xy}(\phi) = \begin{pmatrix} \cos(\phi) & \sin(\phi) & 0 \\ -\sin(\phi) & \cos(\phi) & 0 \\ 0 & 0 & 1 \end{pmatrix}$, and let $k_x = q\cos(\phi), k_y = -q\sin(\phi)$. The new permittivity components after the in-plane rotation can be written as

$$\epsilon_{xx}^t = \frac{1}{2}\left(\epsilon_{xx} + \epsilon_{yy} + \left(\epsilon_{xx} - \epsilon_{yy}\right)\cos(2\phi)\right),$$

$$\epsilon_{yy}^t = \frac{1}{2}\left(\epsilon_{xx} + \epsilon_{yy} + \left(-\epsilon_{xx} + \epsilon_{yy}\right)\cos(2\phi)\right),$$

$$\epsilon_{xy}^t = \left(-\epsilon_{xx} + \epsilon_{yy}\right)\cos(\phi)\sin(\phi).$$

$$\epsilon_{xz}^t = \epsilon_{xz}\cos(\phi),$$

$$\epsilon_{yz}^t = -\epsilon_{xz}\sin(\phi).$$

Maxwell's equations in the new coordinate frame become

$$\widetilde{\mathrm{M}} = \begin{pmatrix} \epsilon_{xx}^t k_0^2 - k_z^2 & \epsilon_{xy}^t k_0^2 & \epsilon_{xz}^t k_0^2 + k_z q \\ \epsilon_{xy}^t k_0^2 & \epsilon_{yy}^t k_0^2 - (k_z^2 + q^2) & \epsilon_{yz}^t k_0^2 \\ \epsilon_{xz}^t k_0^2 + k_z q & \epsilon_{yz}^t k_0^2 & \epsilon_{zz}^t k_0^2 - q^2 \end{pmatrix} \begin{pmatrix} E_x^t \\ E_y^t \\ E_z^t \end{pmatrix} = 0, \quad (3)$$

We can then obtain the solutions for k_z by solving the secular equation $\det(\widetilde{\mathrm{M}}) = 0$. The solutions are separated into two sets, namely: $\boldsymbol{k}^{\mathrm{o}} = (q, 0, k_z^{\mathrm{o}})$, and $\boldsymbol{k}^{\mathrm{e}} = (q, 0, k_z^{\mathrm{e}})$, which represent the wavevectors of ordinary and extraordinary waves, respectively. Their dispersion relations obey

$$q^2 + {k_z^{\mathrm{o}}}^2 = \epsilon_\perp k_0^2, \tag{4.1}$$

$$\frac{(q\sin(\theta)\cos(\phi)+\cos(\theta)k_z^{\mathrm{e}})^2+q^2\sin^2(\phi)}{\epsilon_\parallel} + \frac{(\cos(\phi)\cos(\theta)q-\sin(\theta)k_z^{\mathrm{e}})^2}{\epsilon_\perp} = k_0^2. \tag{4.2}$$

We can then calculate the expressions for the *z*-component of the wavevectors for the eigenmodes in bulk calcite:

$$k_z^{\mathrm{o}} = \pm\sqrt{\epsilon_\perp k_0^2 - q^2}, \tag{5.1}$$

$$k_z^{\mathrm{e}} = \frac{-b\pm\sqrt{b^2-4ac}}{2a}, \tag{5.2}$$

where we defined

$$a = k_0^2(\epsilon_\perp \cos^2(\theta) + \epsilon_\parallel \sin^2(\theta)),$$

$$b = k_0^2(\epsilon_\perp - \epsilon_\parallel)\sin(2\theta)\, q\cos(\phi),$$

$$c = -k_0^4\epsilon_\perp\epsilon_\parallel + k_0^2(\epsilon_\perp \sin^2(\theta) + \epsilon_\parallel \cos^2(\theta))q^2\cos^2(\phi) \ + k_0^2\epsilon_\perp q^2 \sin^2(\phi),$$

in which b is a real number when ohmic losses are neglected. Therefore, if $b^2 - 4ac \leq 0$, k_z^{e} is complex and thus the wave front of the extraordinary wave is oblique with respect to the interface.

Based on Eq. (5.1), we determine the condition by which the ordinary wave propagates in calcite, i.e. when $q < \sqrt{\epsilon_\perp}k_0$, where $q/k_0 = \sqrt{\epsilon_\perp}$ is the light cone of ordinary wave in calcite (blue circle in Fig.1g and Fig. S6a). In general, the necessary condition for the existence of evanescent extraordinary waves is $b^2 - 4ac \leq 0$. Only the passive solution for surface waves is allowed, such that $\Im(k_e) \leq 0$. Thus, the sign in Eq. (5.2) is determined by the sign of a. To study the propagating behavior of the extraordinary wave, we consider the type-I hyperbolic regime ($\epsilon_\perp > 0, \epsilon_\parallel < 0$) and the transparent regime of calcite ($\epsilon_\perp > 0, \epsilon_\parallel > 0$) separately.

(1) In the hyperbolic regime, we investigate the special case when $\theta = 0^\circ$ and $\phi = 0^\circ$, and obtain $k_z^{\mathrm{e}} = -\mathrm{i}\sqrt{\frac{\epsilon_\parallel(q^2-\epsilon_\perp k_0^2)}{\epsilon_\perp}}$ from Eq. (5.2). This expression indicates that when $q^2 < \epsilon_\perp k_0^2$ the extraordinary wave is an evanescent wave confined to the interface; otherwise, it is a propagating wave in the calcite. In summary, in the region $q/k_0 < \sqrt{\epsilon_\perp}$, the ordinary wave is propagating inside calcite while the extraordinary wave in the calcite evanescently decays away from the interface, as indicated by regions 1 and 2 of the momentum space (k-space) in Fig. 1g.

(2) In the transparent regime, we first consider the special case when $\theta = 0^\circ$, obtaining $k_z^{\mathrm{e}} = -\mathrm{i}\sqrt{\epsilon_\parallel(\frac{k_y^2}{\epsilon_\parallel} + \frac{k_x^2}{\epsilon_\perp} - k_0^2)}$. Therefore, when $\frac{k_y^2}{\epsilon_\parallel} + \frac{k_x^2}{\epsilon_\perp} > k_0^2$ the extraordinary wave is an evanescent wave confined to the interface (purple ellipse in Fig. S6a); otherwise, it is propagating wave in the calcite. To sum up, in the region $q/k_0 < \sqrt{\epsilon_\perp}, \frac{k_y^2}{\epsilon_\parallel} + \frac{k_x^2}{\epsilon_\perp} > k_0^2$, the ordinary wave is propagating inside the calcite while the extraordinary wave in the calcite evanescently decays away from the interface, as indicated by regions 1 and 2 of k-space in Fig. S6a.

When the OA of calcite is slanted with respect to the interface, the projected momenta of the bulk mode wavevector on the k_x-k_y plane varies, and it can lead the mode to transition between propagating and evanescent for larger slanted angles θ. To guarantee the evanescent properties of the extraordinary wave in calcite, the condition $b^2 - 4ac \leq 0$ needs to be maintained. Such restriction gives $\Im(k_z^{\mathrm{e}}) < 0$, leading to the constraint $0 \leq k_x^2 \leq \mathrm{q}^2 \frac{\epsilon_\perp \cos(\theta)^2 + \epsilon_\parallel \sin(\theta)^2}{1+2\cos(\theta)^2 \sin(\theta)^2}$. As a result, the range of allowed slanted angle

$$\tan(\theta) \leq \sqrt{-\frac{\epsilon_\perp}{\epsilon_\parallel}}. \tag{6}$$

is obtained.

b. Complex secular equation for leaky polaritons in lossless system

In this sub-section we seek the eigenmode solutions for leaky polaritons at the interface between calcite and air. To get the in-plane momentum dispersion of the LP, we use the solutions for k_z in Eq. (5) to evaluate the electric fields and magnetic fields of the ordinary and extraordinary waves in calcite. Their general forms calculated from Maxwell equations can be chosen as

$$\boldsymbol{E}_{\mathrm{o}}^{\boldsymbol{t}} = [m_{22}^{\mathrm{o}} m_{33}^{\mathrm{o}} - m_{23}^{\mathrm{o}} m_{32}^{\mathrm{o}}, m_{23}^{\mathrm{o}} m_{31}^{\mathrm{o}} - m_{21}^{\mathrm{o}} m_{33}^{\mathrm{o}}, m_{21}^{\mathrm{o}} m_{32}^{\mathrm{o}} - m_{22}^{\mathrm{o}} m_{31}^{\mathrm{o}}]; \quad \boldsymbol{H}_{\mathrm{o}}^{\boldsymbol{t}} = \left[k_z^{\mathrm{o}} e_y^{\mathrm{o}}, -k_z^{\mathrm{o}} e_x^{\mathrm{o}} + q e_z^{\mathrm{o}}, -q e_y^{\mathrm{o}}\right]; \tag{7.1}$$

$$\boldsymbol{E}_{\mathrm{e}}^{\boldsymbol{t}} = [m_{22}^{\mathrm{e}} m_{33}^{\mathrm{e}} - m_{23}^{\mathrm{e}} m_{32}^{\mathrm{e}}, m_{23}^{\mathrm{e}} m_{31}^{\mathrm{e}} - m_{21}^{\mathrm{e}} m_{33}^{\mathrm{e}}, m_{21}^{\mathrm{e}} m_{32}^{\mathrm{e}} - m_{22}^{\mathrm{e}} m_{31}^{\mathrm{e}}];$$

$$\boldsymbol{H}_{\mathrm{e}}^{\boldsymbol{t}} = \left[k_z^{\mathrm{e}} e_y^{e}, -k_z^{\mathrm{e}} e_x^{e} + q e_z^{e}, -q e_y^{e}\right]. \tag{7.2}$$

The fields of TM (p-polarized) wave and TE (s-polarized) wave in the air in the transformed coordinates read

$$\boldsymbol{E}_{\mathbf{p}}^{t} = \left[-k_z^{air}, 0, q\right], \boldsymbol{H}_{\mathbf{p}}^{t} = [0, \epsilon_0, 0]; \tag{8.1}$$

$$\boldsymbol{E}_{\mathbf{s}}^{t} = [0,1,0], \boldsymbol{H}_{\mathbf{s}}^{t} = [k_z^{air}, 0, -q], \tag{8.2}$$

where

$$k_z^{air} = \sqrt{\epsilon_0 k_0^2 - q^2}, \tag{9}$$

is the z component of the wavevector in air. Next, we consider continuous boundary conditions for the tangential fields at the interface

$$\widehat{D}|\psi\rangle = 0, \tag{10}$$

where the vector $|\psi\rangle = \left(a_{\mathrm{o}}, a_{\mathrm{e}}, a_{\mathrm{p}}, a_{\mathrm{s}}\right)^T$ consists of all coefficients of wave components at the interface, and $\widehat{D}$ is

$$\widehat{D} = \begin{pmatrix} e_x^{\mathrm{o}} & e_x^{\mathrm{e}} & -k_z^{\mathrm{air}} & 0 \\ e_y^{\mathrm{o}} & e_y^{\mathrm{e}} & 0 & 1 \\ h_x^{\mathrm{o}} & h_x^{\mathrm{e}} & 0 & k_z^{\mathrm{air}} \\ h_y^{\mathrm{o}} & h_y^{\mathrm{e}} & \epsilon_0 & 0 \end{pmatrix}. \tag{11}$$

We obtain the secular equation by imposing $\det[\widehat{D}] = 0$. For the regular surface mode, the secular equation can be solved in terms of (q, ϕ), or (k_x, k_y), in the real domain $\mathbb{R}$. All z components of the wavevectors in Eqs. (5,9) are imaginary. However, we search for the solutions in the leaky regions 1 and 2 of k-space in Fig. 1g of the main text, which is bounded by the light cone of ordinary (blue line) and extraordinary (purple lines) waves in calcite projected from the bulk isofrequency contour (IFC) for calcite. This implies that, in these regions, p-polarized and s-polarized waves in the air can be evanescent or propagating (leaky) depending on whether they lie outside of the free space light cone (FSLC), and extraordinary waves are evanescent normal to the interface, whereas ordinary waves are leaky into the calcite. Consequently, we never find the solutions (k_x, k_y) in the real domain $\mathbb{R}$ because the time-averaged Poynting vector normal to the interface $\langle \mathrm{S_z} \rangle$ is zero in air. However, $\langle \mathrm{S_z} \rangle \neq 0$ in calcite, except for the position $k_x/k_0 = \sqrt{\frac{\epsilon_\perp \epsilon_0 (\epsilon_0 - \epsilon_\parallel)}{\epsilon_0^2 - \epsilon_\perp \epsilon_\parallel}}$, with $\epsilon_\parallel < 0$ and $\theta = 0^\circ$, where the polariton is evanescent both in air and in calcite (see section II). In order to find all possible solutions in k-space, we look for solutions in the complex domain $\mathbb{C}$, imposing the condition $q = q_\mathrm{r} + i q_\mathrm{i}$ (see section III for the proof). As a consequence, the secular equation turns into:

$$f_\mathrm{r}(q_\mathrm{r}, q_\mathrm{i}, \phi) = \Re(\det[D]) = 0;$$
$$f_\mathrm{i}(q_\mathrm{r}, q_\mathrm{i}, \phi) = \Im(\det[D]) = 0. \tag{12}$$

c. Branch cut and $\langle \mathrm{S_z} \rangle$ diagram

In principle, we can find several complex solutions for the IFC in the parameter space $(q_\mathrm{r}, q_\mathrm{i}, \phi)$ based on Eq. (11). However, some of them are unphysical and thus should be excluded. We must carefully choose the branch cut and sign for the solutions of Eqs. (5,9). The branch cut of $\sqrt{\mathrm{z}}$ is adopted in this work as:

$$\text{Branch cut} := \begin{cases} (-\infty, 0), \text{if } \Re(z) \geq 0 \\ (0, \infty), \text{if } \Re(z) < 0 \end{cases}. \tag{13}$$

As discussed in section I.a, since we search for solutions in the region $q_\mathrm{r}/k_0 < \sqrt{\epsilon_\perp}$, the extraordinary wave in calcite decays evanescently, so that $\Im(k_z^\mathrm{e}) < 0$. The ordinary wave propagates in calcite but the sign of its phase velocity must be determined. Therefore, we have two choices for the sign of $\Re(k_z^\mathrm{o})$. If $\sqrt{\epsilon_0} < \frac{q_\mathrm{r}}{k_0} < \sqrt{\epsilon_\perp}$, p/s-polarized waves in air have to be evanescently damped, hence $\Im(k_z^\mathrm{air}) > 0$. If $\frac{q_\mathrm{r}}{k_0} < \sqrt{\epsilon_0}$, p/s waves in air might also evanescently grow towards positive z due to the waves' leaky properties ($q_\mathrm{i} \neq 0$), such that $\Im(k_z^\mathrm{air}) < 0$. Furthermore, the sign of q_i - the imaginary part of the in-plane momentum - is also not determined yet. Once we define the signs of $\Im(k_z^\mathrm{air})$, $\Re(k_z^\mathrm{o})$ and q_i, the signs of other parameters ($\Re(k_z^\mathrm{air})$, $\Im(k_z^\mathrm{o})$ and $\Re(k_z^\mathrm{e})$) are unambiguously deduced from energy conservation (Maxwell equations). In total, 8 distinguished cases exist, summarized in detail in Table 1. However, not all of them correspond to physical scenarios (green color cases might have physical solutions, and red color cases don't have physical ones as explained in the following).

Table 1. All possible configurations of signs in *z* components of complex wavevectors are listed below.

	Positive phase velocity for o wave: $\Re(k_z^\mathrm{o}) > 0$		Negative phase velocity for o wave: $\Re(k_z^\mathrm{o}) < 0$	
$q = q_\mathrm{r} + iq_\mathrm{i}$	$q_\mathrm{i} > 0$	$q_\mathrm{i} < 0$	$q_\mathrm{i} > 0$	$q_\mathrm{i} < 0$
Decay in the air: $\Im(k_z^\mathrm{air}) > 0$	a: $\Re(k_z^\mathrm{air}) < 0$; $\Im(k_z^\mathrm{o}) < 0$; $\Re(k_z^\mathrm{e}) > 0$.	b: $\Re(k_z^\mathrm{air}) > 0$; $\Im(k_z^\mathrm{o}) > 0$; $\Re(k_z^\mathrm{e}) < 0$.	c: $\Re(k_z^\mathrm{air}) < 0$; $\Im(k_z^\mathrm{o}) > 0$; $\Re(k_z^\mathrm{e}) > 0$.	d: $\Re(k_z^\mathrm{air}) > 0$; $\Im(k_z^\mathrm{o}) < 0$; $\Re(k_z^\mathrm{e}) < 0$.
Grow in the air: $\Im(k_z^\mathrm{air}) < 0$	e: $\Re(k_z^\mathrm{air}) > 0$; $\Im(k_z^\mathrm{o}) < 0$; $\Re(k_z^\mathrm{e}) > 0$.	f: $\Re(k_z^\mathrm{air}) < 0$; $\Im(k_z^\mathrm{o}) > 0$; $\Re(k_z^\mathrm{e}) < 0$.	g: $\Re(k_z^\mathrm{air}) > 0$; $\Im(k_z^\mathrm{o}) > 0$; $\Re(k_z^\mathrm{e}) > 0$.	h: $\Re(k_z^\mathrm{air}) < 0$; $\Im(k_z^\mathrm{o}) < 0$; $\Re(k_z^\mathrm{e}) < 0$.

Next, we utilize the physical boundary condition that requires the continuity of the time-averaged Poynting vector component $\langle S_z \rangle$ at the interface and examine the diagrams of $\langle S_z \rangle$ case by case to exclude the unphysical solutions. To estimate the behavior of $\langle S_z \rangle$ as a function of z, we utilize the following rule of thumb: the directions of $\langle S_z \rangle$ in air and in calcite are determined by the signs of $\Re(k_z^\mathrm{air})$ and $\Re(k_z^\mathrm{o})$, respectively, thanks to the evanescently decayed extraordinary wave in calcite, which contributes to $\langle S_z \rangle$ only near the interface. Based on such rule, we draw the diagrams of $\langle S_z \rangle$ for all cases in Fig.S2. The cases considered in panels (b,c,e,h) in Fig. S2 are physically possible because the continuity of $\langle S_z \rangle$ at the interface is satisfied. Henceforth, we eliminate the unphysical solutions in Fig. S2 (a,d,f,g) without entering the details of the calculation. Note that when ohmic losses are ignored, the conservation of time reversal symmetry implies that cases (b,c), and cases (e,h) correspond to the time reversal with each other.

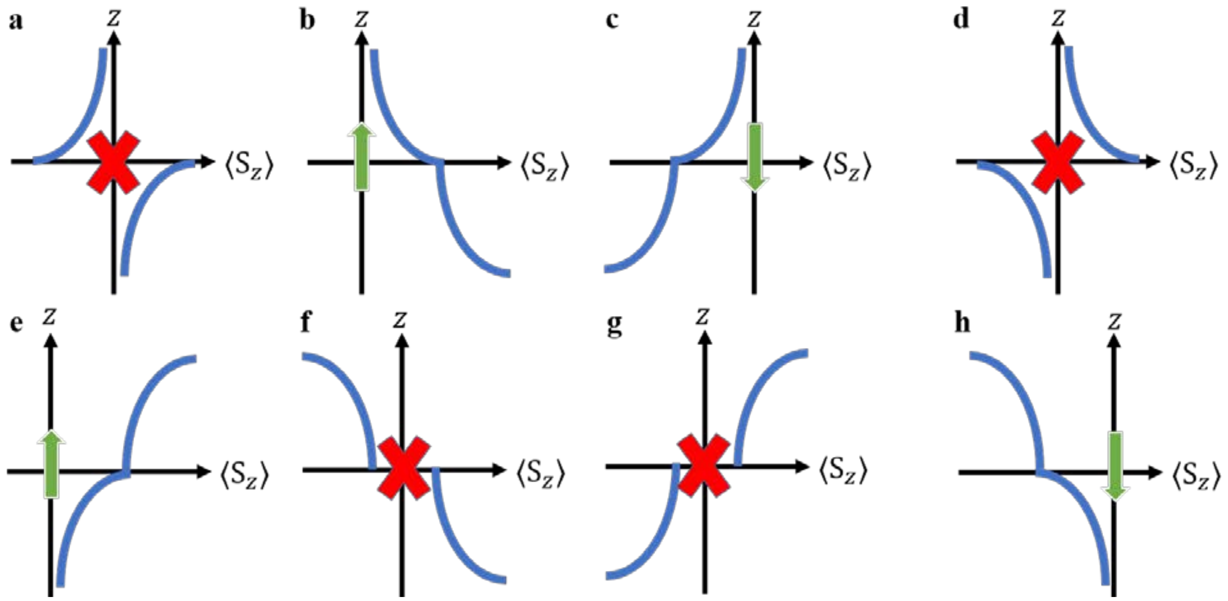

Figure S2: The diagrams of the time-average Poynting vector in z direction $\langle S_z \rangle$ for cases a-h listed on Table 1. The red cross marks indicate that continuity of Poynting flux cannot be satisfied. The green arrows show the direction of Poynting flux along the z direction.

d. Field distributions and Poynting vectors

To find the eigenmodes distribution of LP in the real space, we evaluate the fields in rectangular coordinates by means of the following rotation

$$\boldsymbol{E_i}(k_x, k_y) = \hat{R}_{xy}(\phi)\boldsymbol{E_i^t}, \tag{14.1}$$

$$\boldsymbol{H_i}(k_x, k_y) = \hat{R}_{xy}(\phi)\boldsymbol{H_i^t}, \tag{14.2}$$

where the index i represents p/s-polarized waves in air or ordinary/extraordinary waves in calcite. Subsequently, we set the coefficient of the p wave in Eq. (9) equal to one (other choices of the coefficient can be made and it does not alter the conclusions), and evaluate the remaining coefficients by the reduced continuous boundary conditions of the fields parallel to the interface

$$\widehat{D}'|\psi\rangle' = |\varphi_{\mathrm{p}}\rangle', \tag{15}$$

where

$$|\psi\rangle' = (a'_{\mathrm{o}}, a'_{\mathrm{e}}, a'_{\mathrm{s}})^T,$$

$$|\varphi_{\mathrm{p}}\rangle' = \left(e_y^{\mathrm{p}\prime}, h_x^{\mathrm{p}\prime}, h_y^{\mathrm{p}\prime}\right)^T,$$

and

$$\widehat{D}' = \begin{pmatrix} e_y^{\mathrm{o}\prime} & e_y^{\mathrm{e}\prime} & -e_y^{\mathrm{s}\prime} \\ h_x^{\mathrm{o}\prime} & h_x^{\mathrm{e}\prime} & -h_x^{\mathrm{s}\prime} \\ h_y^{\mathrm{o}\prime} & h_y^{\mathrm{e}\prime} & -h_y^{\mathrm{s}\prime} \end{pmatrix}. \tag{16}$$

The elements in $\widehat{D}'$ are the fields in rectangular coordinates. Note that other choices of tangential fields for the boundary conditions produce the same results as those from Eq. (16), except the fields might be different up to an overall phase factor. The real-space distributions of electric fields and magnetics fields are evaluated after calculating the coefficients from Eq. (15) as

$$\begin{aligned}\boldsymbol{E}_{\text{air}}(\boldsymbol{r}) &= (\boldsymbol{E}_{\text{p}} + a'_{\text{s}}\boldsymbol{E}_{\text{s}})e^{i(k_x x + k_y y + k_z^{\text{air}} z)};\\ \boldsymbol{H}_{\text{air}}(\boldsymbol{r}) &= (\boldsymbol{H}_{\text{p}} + a'_{\text{s}}\boldsymbol{H}_{\text{s}})e^{i(k_x x + k_y y + k_z^{\text{air}} z)};\\ \boldsymbol{E}_{\text{calcite}}(\boldsymbol{r}) &= (a'_{\text{o}}\boldsymbol{E}_{\text{o}}e^{ik_z^{\text{o}} z} + a'_{\text{e}}\boldsymbol{E}_{\text{e}}e^{ik_z^{\text{e}} z})e^{i(k_x x + k_y y)};\\ \boldsymbol{H}_{\text{calcite}}(\boldsymbol{r}) &= (a'_{\text{o}}\boldsymbol{H}_{\text{o}}e^{ik_z^{\text{o}} z} + a'_{\text{e}}\boldsymbol{H}_{\text{e}}e^{ik_z^{\text{e}} z})e^{i(k_x x + k_y y)}.\end{aligned} \tag{17}$$

Then, the time-averaged Poynting vector for the regions of air/calcite are evaluated as $\langle \boldsymbol{S}_{\text{air/calcite}} \rangle = (1/2)\Re(\boldsymbol{E}_{\text{air/calcite}} \times \boldsymbol{H}^*_{\text{air/calcite}})$.

Having established the complex eigenvalue equation and the formulae for the various physical quantities, we first examine the fields and Poynting vectors of the conventional surface hyperbolic polaritons (s-HPs) and ghost hyperbolic polaritons (g-HPs) in the upper Reststrahlen band of calcite (as shown in Fig. S1), at frequency $\omega = 1470\ \text{cm}^{-1}$, with $\theta = 0^\circ$ and $\theta = 23.3^\circ$, respectively. The in-plane dispersion of the surface polaritons (red curves) and bulk polaritons (blue curves) for the cases $\theta = 0^\circ$ and $\theta = 23.3^\circ$ are shown in Fig. S3(a,c). The electric fields (colormap) and Poynting vectors (arrowheads) in real space are displayed in Fig. S3(b,d), respectively. Throughout the manuscript, the colormap ranges of E_x and E_y distributions for the same eigenmode are identical for better comparison, but they are not necessarily the same for different modes. As we expect, the fields decay evanescently away from the interface and Poynting vectors are parallel to the interface for both s-HPs and g-HPs. However, while the wavefront of the regular surface polariton is perpendicular to the interface (Fig. S3b), both electric fields E_x and E_y of g-HPs exhibit oblique wavefronts, which decay evanescently away from the interface, and their Poynting vectors are strictly parallel to the interface, confirming their bound nature (Fig. S3d). Moreover, the behaviors of phase and Poynting vectors of the surface polaritons are independent of the field polarization, since E_x and E_y exhibit alike field distributions as shown in Fig. S3b,d.

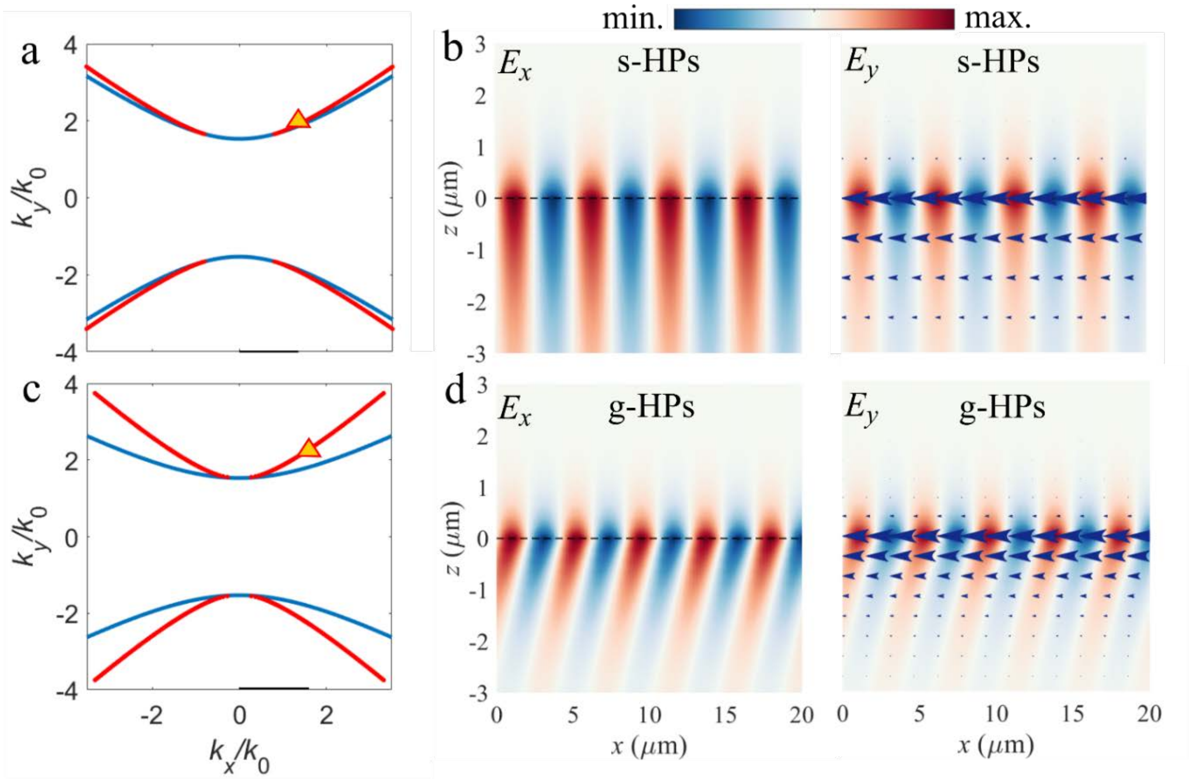

Figure S3: Eigenmode study for upper bands at frequency $\omega = 1470\ \mathrm{cm}^{-1}$. (a) IFC of surface hyperbolic polaritons (s-HPs) with $\theta = 0°$. (b) Electric fields E_x and E_y distributions and Poynting vector flows in real space of s-HPs. (c) IFC of ghost hyperbolic polaritons (g-HPs) with $\theta = 23.3^{\circ}$. (d) Electric fields E_x and E_y distributions and Poynting vector flows in real space of g-HPs. The positions of s-HPs and g-HPs are marked by triangles shown in a. c.

Next, we study the case of LP in the type-I hyperbolic regime, at frequency $\omega = 887\ \mathrm{cm}^{-1}$ and $\theta = 0^{\circ}$, where the OA of calcite aligned in the interface, and plot the real-space field distributions and Poynting vectors of LP located outside and inside FSLC, separately, and their positions are indicated in Fig. 1g of the main text. The field distributions and Poynting vectors in Fig. S4(a,b) are shown in both *x*-*z* cross-section and *y*-*z* cross-section of the 3D spatial domain. The field distributions of LP in real space are polarization dependent and behave distinctly outside and inside the FSLC, due to the anisotropic response of calcite. When the LP propagate in the direction close to that of the OA projected onto the *x*-*y* plane (outside the FSLC), the E_x component of LPs shown in Fig. S4a, has a similarly confined spatial profile as HPs in Fig. S3b, and is dominant in the LP in all spatial regions. While E_y shows tilted radiative leakage with marginal magnitude, as its momentum is compatible with the ordinary bulk wave in calcite. In this case LP are majorly composed of evanescent surface waves in the form of extraordinary waves in calcite and p-polarized waves in air, and both their wave front and Poynting vectors are (almost) parallel to the interface, alike the conventional s-HPs in Fig. S3(a,b). Interestingly, the LP also demonstrate oblique wave fronts in calcite associated with their leaky dispersion, distinct from the tilted wavefronts of g-HPs, which require a tilted optical axis. When the LP are inside the FSLC (Fig. S4b), since they are substantially composed of the propagating waves both in air and in calcite,

where the electric field components of ordinary waves in calcite do not contain E_x, E_y is dominant in all spatial regions, and E_x is zero in the calcite (Fig. S4b), which is generally true for the cases at different frequencies and $\theta = 0^\circ$. As a result, the Poynting vectors of LP are oblique relative to the interface, and flow into the calcite in all cross sections. The continuous flow of Poynting vector implies that LP inside the FSLC are reminiscent of the Brewster angle, at which a p-polarized wave impinging on an interface is fully transmitted from one medium to the other (we will discuss this in detail in the section dedicated to the symmetry point $k_x = 0$).

We next study LP at frequency $\omega = 887\ \text{cm}^{-1}$ and $\theta = 23.3^\circ$. LP in this case share most of the features of those in the last example, excluding the two described in the following. When the propagation direction of LP is close to the direction of the OA projected in the *x*-*y* plane (outside FSLC), their surface wave front is slanted with respect to the interface, and the Poynting flux is (almost) parallel to the interface and is confined near the interface, as shown in Fig. S5a, which are reminiscent of g-HPs discussed before. When LP are inside the FSLC, both E_x and E_y are characterized by tilted wavefronts, the dominant ordinary waves in calcite also contain an E_x component which stems from the tilted OA, although it is much weaker than E_y in calcite (see lower spatial regions in Fig. S5b).

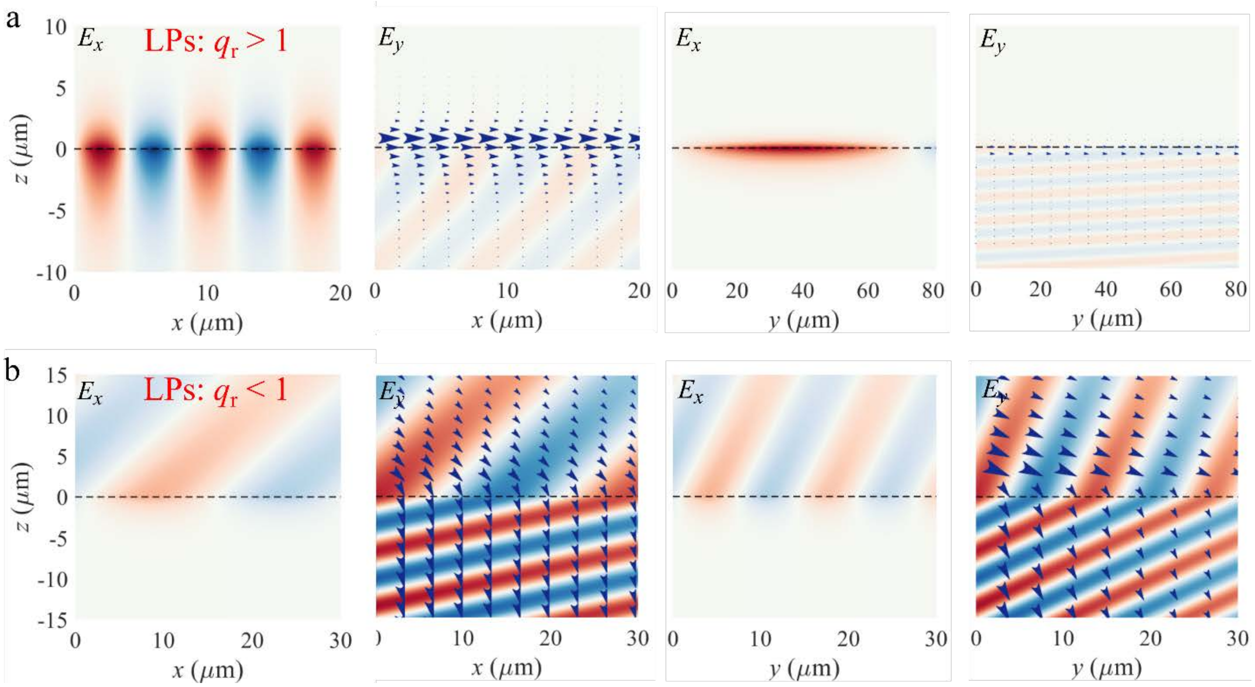

Figure S4: Field distributions and Poynting vectors of LPs at $\omega = 887\text{cm}^{-1}$, $\theta = 0^\circ$ in real space. a. Fields of LP outside FSLC ($q_r > 1$). b. Fields of LP inside FSLC ($q_r < 1$). E_x and E_y fields and Poynting vectors (arrows) in *x*-*z* cross-section of the interface are shown in the left two panels and in *y*-*z* cross-section in the right two panels.

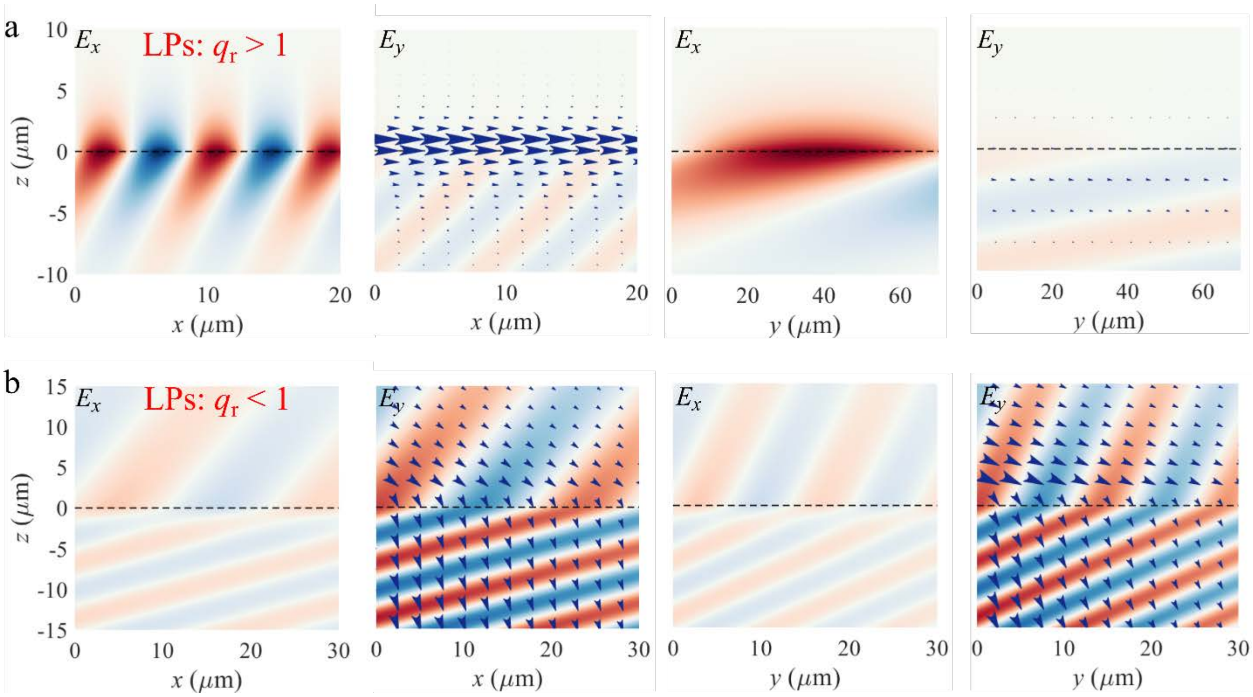

Figure S5: Field distributions and Poynting vectors of LPs at $\omega = 887\text{cm}^{-1}$, $\theta = 23.3^{\circ}$ in real space. a. Fields of LP outside FSLC($q_r > 1$). b. Fields of LPs inside FSLC($q_r < 1$). E_x and E_y fields and Poynting vectors in *x*-*z* cross-section of the interface are shown in the left two panels and in *y*-*z* cross-section in the right two panels.

As we mention in section I.a, LPs also exist in the transparent regime of the lower Reststrahlen band, where $\epsilon_{\perp} > 0$ and $0 < \epsilon_{\parallel} < \epsilon_{\text{air}}$. Similar to the type-I hyperbolic regime described in the main text, in Fig. S6 we show the IFCs of both bulk modes in 3D k-space and their corresponding projected IFCs in 2D k-space (in-plane) at frequency $\omega = 890\ \text{cm}^{-1}$. In this regime, calcite supports modes of isotropic ordinary waves, whose dispersion is described by the outer sphere in Fig. S6a (top panel), and extraordinary waves with elliptical dispersion, represented by the purple ellipsoid, whose main axis is aligned along the OA. The isotropic IFC of the radiation modes in free-space is represented by the inner white sphere. These surfaces represent the three light cones for modes traveling along the calcite-air interface, projected onto the k_x-k_y plane in the same panel for different values of θ. The modes outside of the spherical and ellipsoidal contours are guided at the interface. Oppositely, the modes living inside these contours are propagating in calcite and in air. Accordingly, the in-plane *k*-space can be divided into four regions described in the main text as well: region 1 indicated by a yellow shade in supplementary Fig. 6a corresponds to modes inside the FSLC (white circle) and the light cone of the ordinary waves in calcite (blue circle), but they are outside the light cone of extraordinary waves in calcite (purple ellipse). Hence, in this region we expect modes that leak energy both in air and in ordinary waves of calcite. Similarly, in region 2 (light blue color in Fig. S6a) modes are leaky in calcite in the form of ordinary waves but evanescently decay in free space; in region 4 (grey color in Fig. S6a) the modes evanescently decay away from the interface in both free space and calcite; in region 5 (cyan color in Fig. S6a), the modes are leaky in calcite and evanescent in free space, in region 6 (pink color in Fig. S6a) the modes are leaky both in air and in calcite. Similar to type-I hyperbolic regime, we find the complex

eigenmode solutions in leaky region 1 and 2, and obtain the dispersion of LP, characterized by a lenticular shape in k-space shown by red curves in supplementary Fig. 6a. Interestingly, the in-plane dispersion is not a close contour and it is cut by the intersection with the elliptical IFCs of extraordinary waves in the bulk of region 2. As we increase the angle θ between the OA and the interface, the elliptical light cone of the extraordinary wave is increasingly tilted. Thus, its projection in the k_x-k_y plane shrinks in the x direction, pushing the LP dispersion towards the origin in the k_x direction, until the projected ellipse contour enters LLFS. In this regime, the extraordinary wave in calcite along k_x cannot be guided any longer by the interface, and LP are no longer supported.

Next, we plot the field distributions and Poynting vectors of LP at $\theta = 23.3^{\circ}$ inside and outside of the FSLC (Fig. S6b,c). Both the E_x and E_y components of LP are shown in Fig. S6b, when their in-plane wavenumber is outside the FSLC and away from the direction of OA projected along k_x direction (Fig. S6a, triangular dot denoted by the letter b), has hybridized character between a confined extraordinary wave and a leaky ordinary wave, and both show tilted wave fronts. The Poynting vectors are slightly skewed toward the bulk of calcite, indicating that the evanescent extraordinary wave is the major component of LP. When LP are inside the FSLC (the position is shown in Fig. S6a, triangular dot denoted by letter c), both E_x and E_y are characterized by tilted wavefronts, and the Poynting vectors are tilted more into calcite compared to the ones outside FSLC, implying that the leaky ordinary wave dominates the fields in LP. Similar to the main text, we conclude that the hybrid features of leaky ordinary waves and evanescent extraordinary waves are strongly dependent on the in-plane wavevector.

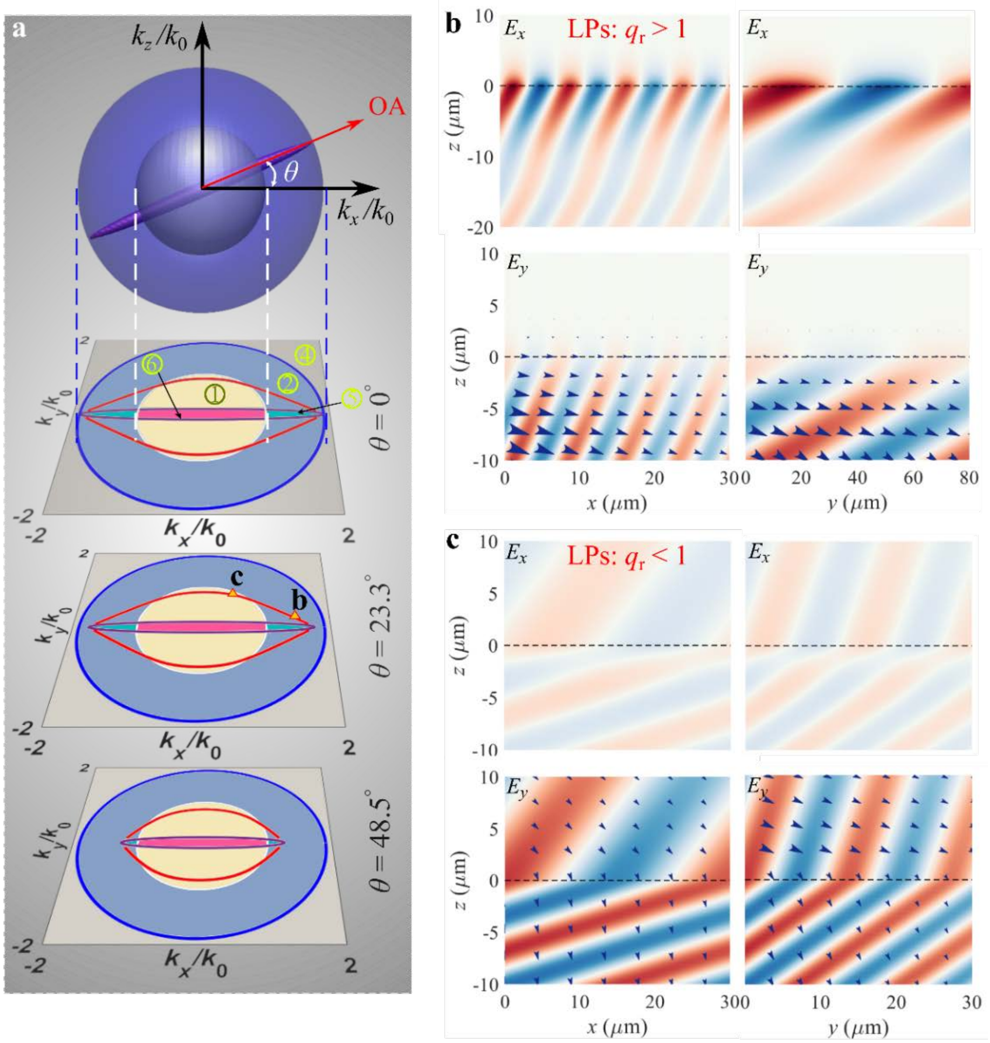

Figure S6: LP in the transparent regime. a. dispersion of LP at $\omega = 890\ \mathrm{cm}^{-1}$ with different tilted angles of OA. **b,c.** Field distributions and Poynting vectors of LP at $\omega = 890\mathrm{cm}^{-1}$, $\theta = 23.3^{\circ}$ in real space. **b.** Fields of LP outside FSLC on *x*-*z* and *y*-*z* cross sections. **c.** Fields of LP inside FSLC on *x*-*z* and *y*-*z* cross sections.

II. Solutions of leaky polaritons at symmetry points

a. $\boldsymbol{k_y = 0}$

When the polaritons propagate in the plane of the OA, it indicates the wavevector component $k_y = 0$, or equivalently, $\phi = 0^{\circ}$. The ordinary wave in calcite thus becomes an s-polarized wave and the correspondent eigenfields turn into:

$$\boldsymbol{E}_{\mathrm{o}} = [0,1,0],$$

$$\boldsymbol{H}_{\mathrm{o}} = [-ik_z^{\mathrm{o}}, 0, -q].$$

Meanwhile, the extraordinary wave becomes a p-polarized wave and the fields are:

$$\boldsymbol{E}_{\mathrm{e}} = \left[ik_z^{\mathrm{e}}q, 0, \epsilon_{\parallel} + k_z^{\mathrm{e}2}\right],$$
$$\boldsymbol{H}_{\mathrm{e}} = [0, q\epsilon_{\parallel}, 0],$$

where $k_z^{\mathrm{e}} = -\mathrm{i}\sqrt{\frac{\epsilon_{\parallel}(q^2-\epsilon_{\perp}k_0^2)}{\epsilon_{\perp}}}$ and k_z^{o} the same as in Eq. (5.1). To find the solutions, we match the tangential fields at the interface for the evanescent p-polarized wave in air and evanescent extraordinary wave in the calcite and obtain the following expression

$$q^2 = \frac{\epsilon_{\perp}\epsilon_0(\epsilon_0-\epsilon_{\parallel})}{\epsilon_0^2-\epsilon_{\perp}\epsilon_{\parallel}}k_0^2 \geq 0, \tag{18}$$

which indicates the in-plane momentum $(k_x, k_y) = k_0(\sqrt{\frac{\epsilon_{\perp}\epsilon_0(\epsilon_0-\epsilon_{\parallel})}{\epsilon_0^2-\epsilon_{\perp}\epsilon_{\parallel}}},0)$ when $\epsilon_{\parallel} < 0,\ \theta = 0^{\circ}$. Note that when the symmetry point approaches the light line of free space $k_x \to k_0$ with finite value $\epsilon_{\perp} > 1$, $\epsilon_{\parallel} \to -\infty$. This implies that the LP exist throughout the type-I hyperbolic regime.

b. $\boldsymbol{k_x = 0}$

When the polaritons propagate in the direction normal to the plane of the OA, it requires the wavevector component $k_x = 0$, or equivalently, $\phi = 90^{\circ}$. In this case, the ordinary wave in the calcite instead becomes a p-polarized wave and the fields are written as:

$$\boldsymbol{E}_{\mathrm{o}} = [ik_z^{\mathrm{o}}, 0, q],$$
$$\boldsymbol{H}_{\mathrm{o}} = [0, \epsilon_{\perp}, 0].$$

The extraordinary wave changes to an s-polarized wave and the corresponding fields are:

$$\boldsymbol{E}_{\mathrm{e}} = [0,1,0\ ],$$
$$\boldsymbol{H}_{\mathrm{e}} = [-ik_z^{\mathrm{e}}, 0, -q],$$

in which $k_z^{\mathrm{e}} = -\mathrm{i}\sqrt{q^2 - \epsilon_{\parallel}k_0^2}$ and k_z^{o} is the same as in Eq. (5.1). Since we find the solution in the FSLC, we impose the continuous boundary conditions for the propagating p-polarized wave in free space and the propagating ordinary wave in the calcite and get the solution at symmetry point at

$$q^2 = \frac{\epsilon_{\perp}\epsilon_0}{\epsilon_0+\epsilon_{\perp}}k_0^2 \geq 0. \tag{19}$$

Interestingly, the solution for the leaky mode in the FSLC does not depend on the value of $\epsilon_{\parallel}$, which implies that the tilted angle θ of the OA does not affect the solution at $(k_x, k_y) = k_0\left(0, \sqrt{\frac{\epsilon_{\perp}\epsilon_0}{\epsilon_0+\epsilon_{\perp}}}\right)$. Interestingly, this point corresponds to the Brewster angle for which propagating *p*-polarized waves from air couple with the ordinary wave in calcite with unitary transmittance.

III. Directional hybridization and in-plane damping rate

In order to describe the leaky properties and understand the peculiar features of LPs, we examine the imaginary component of the in-plane momentum q_{i} for $\theta = 0^{\circ}$ in one *k*-space quadrant (other quadrants are duplicates by symmetry). While this analysis focuses for simplicity on the symmetric scenario $\theta = 0^{\circ}$, similar features can be predicted for slanted optical axis. Intriguingly, although

material loss is absent, q_{i} (red dashed line in Fig. S7a) is nonzero everywhere, except at the symmetry points $k_y = 0$ ($\phi = 0$) and $k_x = 0$ ($\phi = \pi/2$). As discussed in the Sec. II, at these two symmetry points the contribution of either ordinary or extraordinary waves in LPs, $\tilde{a}_{\mathrm{o}}$ or $\tilde{a}_{\mathrm{e}}$, is zero, as shown by the purple and green curves in Fig. S7a. More specifically, at the symmetry point $\phi = 0$, $\tilde{a}_{\mathrm{o}} = 0$ and $\tilde{a}_{\mathrm{e}} = 1$, indicating that the polariton in calcite consists solely of the extraordinary wave, bound to the interface. In this regime, the LP resumes the properties of a regular surface polariton and it is not radiative ($q_{\mathrm{i}} = 0, \Re(k_z^{\mathrm{air}}) = 0$), with in-plane momentum $(k_x, k_y) = (\sqrt{\frac{\epsilon_\perp \epsilon_0 (\epsilon_0 - \epsilon_\parallel)}{\epsilon_0^2 - \epsilon_\perp \epsilon_\parallel}}, 0)$ (when $\epsilon_\parallel < 0$). As soon as the LP deviates from $\phi = 0$, symmetry is broken and $\tilde{a}_{\mathrm{o}} \neq 0$, contributing to radiation leakage. In turn, the LP radiates also in air due to its complex wave number ($q_{\mathrm{i}} \neq 0, \Re(k_z^{\mathrm{air}}) \neq 0$), ensuring a continuous energy flow at the interface. The ordinary wave contribution increases as the LPs move further away from $\phi = 0$, and both q_{i} and $\Re(k_z^{\mathrm{air}})$ accordingly increase (dashed red and solid blue curves in the grey region of Fig. S7a), causing more radiation leakage. As commonly done in leaky-wave theory[2], we can define a modified free-space light cone (m-FSLC) $q_{\mathrm{r}}^0 = \sqrt{k_0^2 + q_i^2}$ as the transition between the two regions (black dashed line in Fig. S7a), corresponding to the cut-off past which the LP loses its efficient radiation properties, as $\Re(k_z^{\mathrm{air}}) = \Im(k_z^{\mathrm{air}})$. Within the m-FSLC, free-space radiation leakage is more pronounced and directional.

At the other symmetry point $\phi = \pi/2$, $\tilde{a}_{\mathrm{o}} = 1$ and $\tilde{a}_{\mathrm{e}} = 0$, i.e., the LP is dominated by the ordinary wave. In this extreme, the polariton is compatible with radiation in both air and calcite, with in-plane momentum $(k_x, k_y) = (0, \sqrt{\frac{\epsilon_\perp \epsilon_0}{\epsilon_0 + \epsilon_\perp}})$. Clearly at this singularity the LP loses any confinement to the interface. As we depart from $\phi = \pi/2$, the extraordinary wave emerges ($\tilde{a}_{\mathrm{e}} \neq 0$) and as a consequence both q_{i} and $\Im(k_z^{\mathrm{air}})$ (dashed red and dashed blue curve in the yellow region of Fig. S7a) increase, confining a portion of the LP energy at the interface.

To characterize the damping features of LPs based on the eigenmodes analysis, we define the in-plane damping rate as follows:

$$\gamma(k_x, k_y) = q_{\mathrm{i}}/q_{\mathrm{r}}. \tag{20}$$

This quantity describes the propagation length of the LP in the *x*-*y* plane. The impact of material loss on the LP features can be assessed by evaluating the in-plane damping rate ($\gamma_{\mathrm{tot}} = q_i/q_r$) with dissipation in calcite, which describes the inverse propagation length of LPs in the *x*-*y* plane. The damping rate γ_{rad} without material loss, shown by the blue solid curve in Fig. S7b, is associated to the radiation loss, consistent with q_{i} in Fig. S7a: it is zero at the symmetry points for which there is no radiation, and maximum near the m-FSLC. After including realistic calcite loss, the damping rate γ_{tot} of LPs (orange curve in Fig. S7b) consists of two parts: $\gamma_{\mathrm{tot}} = \gamma_{\mathrm{rad}} + \gamma_{\mathrm{dis}}$, where γ_{dis} is the damping rate due to the material loss. As denoted by the cyan curve in Fig. S7b, γ_{dis} is barely affected at $\phi = \pi/2$, and it gradually increases as the LPs move closer to the symmetry point at $\phi = 0$. Since extraordinary waves contribute more in this region, at this point

the LPs are mostly confined at the interface and experience stronger in-plane dissipation in the presence of material loss.

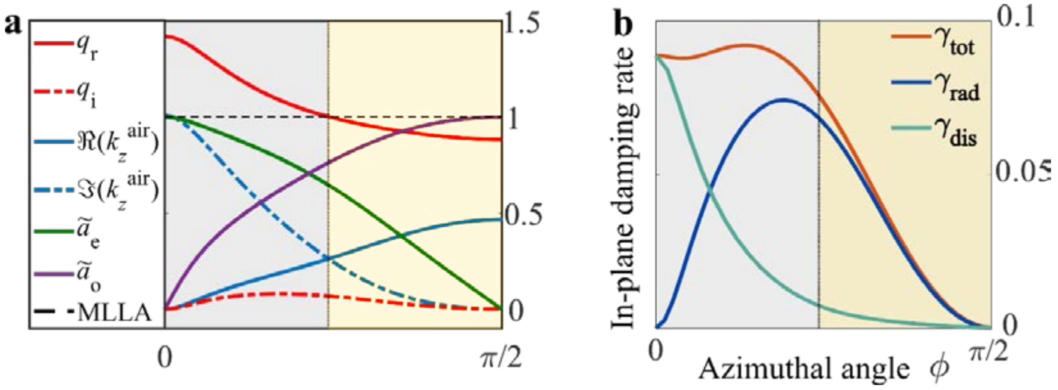

Figure S7. Directional hybridization and damping rate of LPs. a. Phase transition of LPs in a quadrant of k-space. b. In-plane damping rate $\gamma_{\mathrm{tot}}(\gamma_{\mathrm{rad}})$ of LPs with (without) loss in calcite in a quadrant of k-space. The LPs study in a.b. are for the case at frequency $\omega = 887\mathrm{cm}^{-1}$ and $\theta = 0^{\circ}$.

IV. Explanation for the parallel condition of $\boldsymbol{q}_{\mathbf{i}}$ and $\boldsymbol{q}_{\mathbf{r}}$

We first consider the case when the material loss is ignored. Since we search for the solutions of LPs in region (1) and region (4) of Fig.1(g), the ordinary wave in calcite is a propagating wave, thus the radiative loss of the LPs is majorly contributed by the component of ordinary wave in calcite. Since the dispersion of ordinary wave is isotropic represented by a circle in *kx*-*ky* momentum space, the radiation loss of LP follows the direction of its momentum, equivalently, $\boldsymbol{q}_{\mathbf{i}}$ follows the direction of $\boldsymbol{q}_{\mathbf{r}}$, thus ϕ should be real-valued. When the material loss is taken into accounted, the phase velocity and group velocity are in general different, consequently $\boldsymbol{q}_{\mathbf{i}}$ and $\boldsymbol{q}_{\mathbf{r}}$ are in different direction. However, we learn from the near-field simulation that IFCs of LPs are closed contour and the directions of group velocity and phase velocity deviate not much, it is still a good approximation that $\boldsymbol{q}_{\mathbf{i}}$ and $\boldsymbol{q}_{\mathbf{r}}$ of LPs follow the same direction in most of the momentum space.

V. Experimental data from Otto-type polariton spectroscopy measurement

a. Data for calcite with $\theta = 23.3^{\circ}$

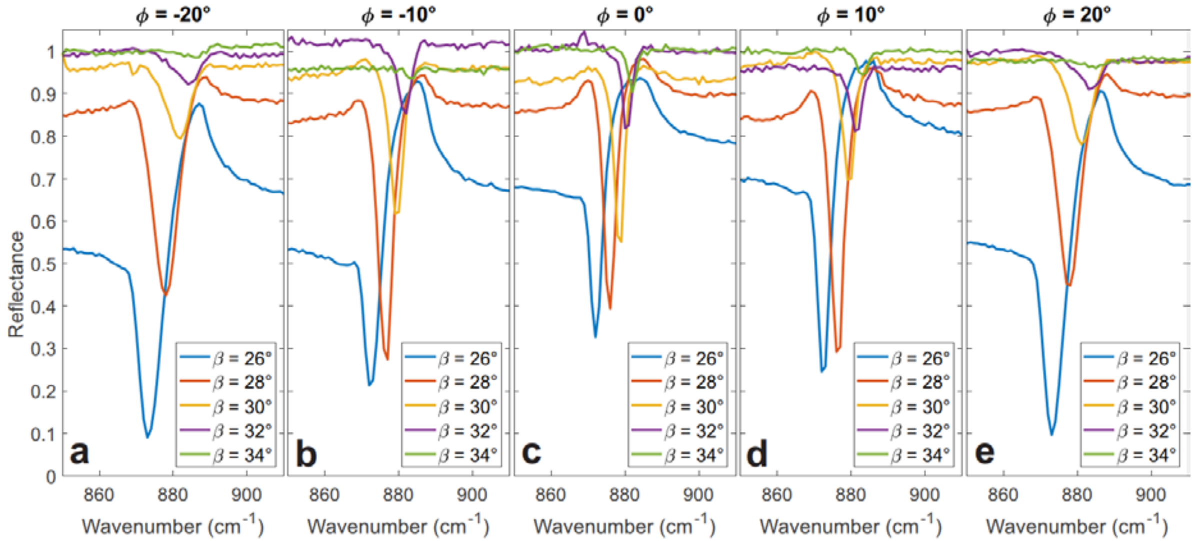

Figure S8. Experimental data sets for Otto-type prism coupling measurement of calcite (100). (a-e) Experimental Otto reflectance spectra at fixed azimuthal angles ϕ's for the calcite (100) sample, taken adjacent to the crystal's optical axis. For each azimuth angle, the spectra were measured at five different incidence angles β: $\beta = 26°, 28°, 30°, 32°, 34°$. The optical axis of the sample is slanted by an angle of $\theta = 23.3^{\circ}$ with respect to the surface. For all the measurements, the prism-sample gap was kept fixed at $\mathrm{d_{gap}} = 4\ \mu m$.

b. Data for calcite with $\theta = 48.5^{\circ}$

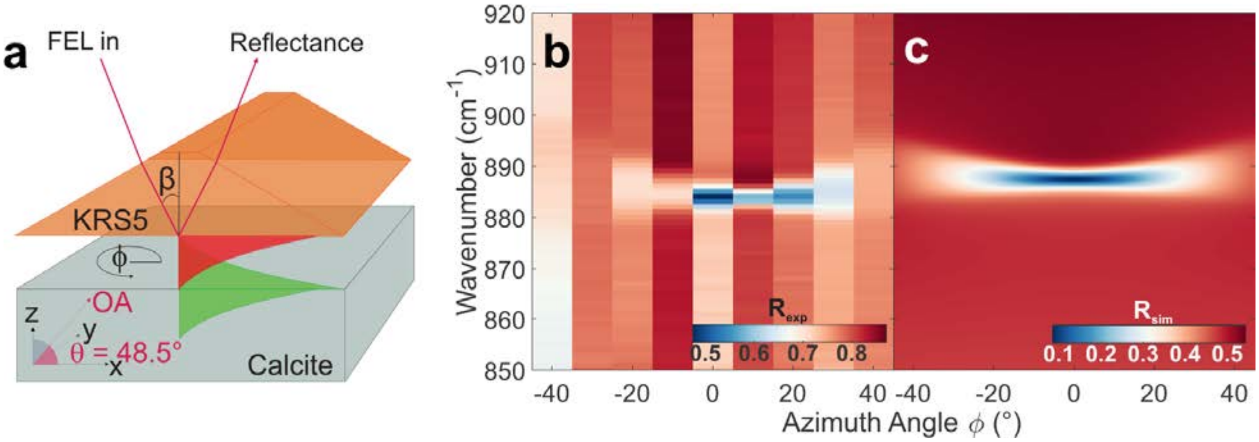

Figure S9. Otto-type prism coupling measurement for the experimental observation of the azimuthal dispersion of LPs in calcite (104). (a) Sketch of the Otto-type prism coupling geometry applied for the excitation and detection of surface phonon polaritons, with details of the investigated sample. (b) Azimuthal dispersion extracted from experimental data and (c) TMM simulations for calcite (104) in which the optical axis is slanted by $\theta = 48.5^{\circ}$ with respect to the surface (as displayed in a). The simulations were performed by taking into account the presence

of a KRS5 prism when setting up the material system, and calculating for each configuration the corresponding reflectance spectrum. The incidence angle is fixed at $\beta = 27°$. The gap between prism and sample is fixed at $d_{gap} = 4$ µm.

c. Data for SiO2

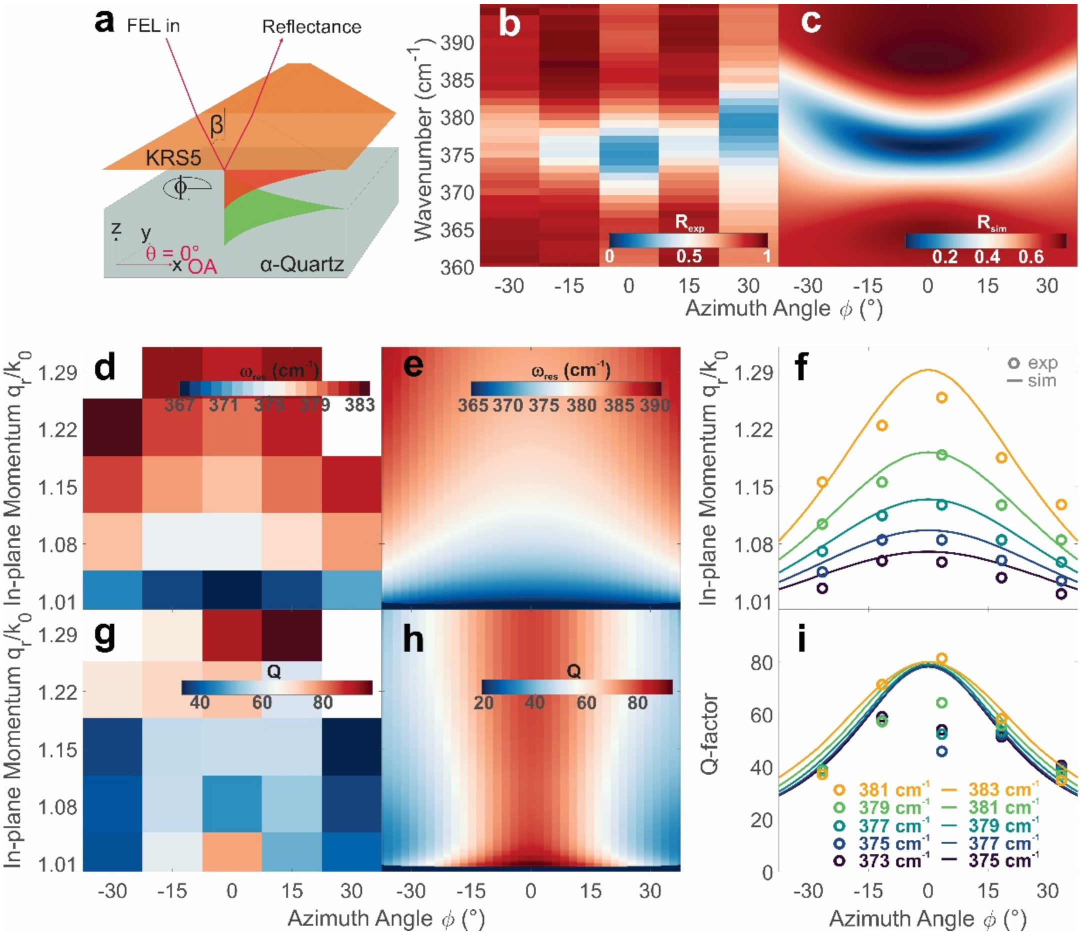

Figure S10. Otto-type prism coupling measurement for the experimental observation of LPs on c-cut α-Quartz (SiO2). (a) Sketch of the Otto-type prism coupling geometry applied for the excitation and detection of surface phonon polaritons, with details of the investigated sample. (b) Azimuthal dispersion extracted from experimental data and (c) TMM simulations for c-cut α-Quartz (SiO2). The c-axis (which coincides with the optical axis) is oriented along the x-direction and lies on the sample surface (as displayed in a). The simulations were performed by taking into account the presence of a KRS5 prism when setting up the material system, and calculating for each configuration the corresponding reflectance spectrum. The in-plane momentum is fixed as $\frac{q}{k_0} = 1.08$ (corresponding to $\beta = 28°$). At this incidence angle (i.e., in the vicinity of the light line in air), the gap between prism and sample is fixed at $d_{gap} = 12$ µm. (d) Experimental and (e) simulated polariton resonance frequency map. The polariton resonance frequencies in the simulated maps are derived from the imaginary part of the reflection coefficients for p-polarized light Im(rpp). Here, the gap is fixed at $d_{gap} = 12$ µm for $\beta = 26°, 28°$, and at $d_{gap} = 9$ µm for $\beta = 30°, 32°, 34°$, in order to achieve optimal efficiency in each configuration. (f) Simulated (lines) and experimental (circles) IFCs at multiple frequencies, demonstrating the lenticular dispersion of LPs. (g) Experimental and (h) simulated LP Q-factors as a function of the in-plane momentum. (i)

Simulated (lines) and experimental (circles) LP Q-factors along the IFCs at multiple frequencies. In Fig. S16 (f,i), the experimental data (circles) are shifted according to the absolute azimuth offset $\Delta\phi = 3.4°$, that was computed as explained in the Methods of the main text.

VI. Near field imaging experiment

a. Experiment for calcite with $\boldsymbol{\theta = 23.3^{\circ}}$

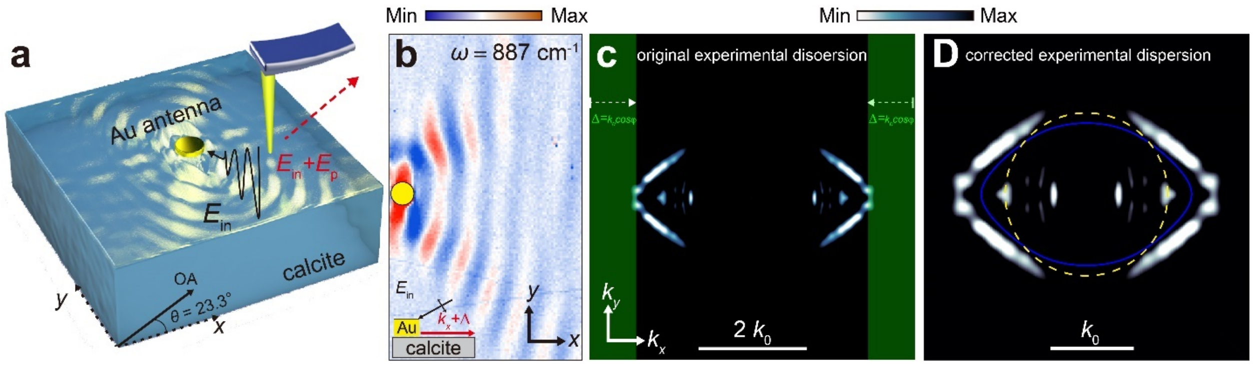

Figure S11. Illustration of SNOM measurements and comparison between experimental near-field images of disk-launched LPs and corresponding theory of in-plane dispersion. a. Schematics of SNOM measurement of directional propagation of LPs. A metallic tip of s-SNOM records the interference field $E_{\mathrm{in}}+E_{\mathrm{p}}$ when the sample is scanned, yielding a near-field optical image with nanoscale resolution. The angle of OA with respect to the calcite surface is $\theta = 23.3^{\circ}$. b. The experimental results were recorded on calcite at ω = 887 cm^{-1}. The schematic in bottom shows the basic mechanism of disk-launched field at oblique illumination. The record near-field distribution is the interference field $E_{\mathrm{in}}+E_{\mathrm{p}}$ thus rendering larger record polariton momentum along x direction, which is similar with reference. c. Corresponding FT of field in b. The measured IFCs of wave vectors illustrated in (b) are shifted by $\pm k_0 \cos\varphi$ along x direction. To experimentally resolve the intrinsic dispersion here, we correct the measured k_x to $k^{\wedge}_x = k_x \pm k_0 \cos\varphi$ for left and right part of the dispersion. (The incident φ is 30° along x direction). d. The comparison between corrected experimental dispersion and corresponding theoretical in-plane dispersion. The yellow dashed line is free-space light cone and the blue solid line is the intrinsic in-plane dispersion of LPs.

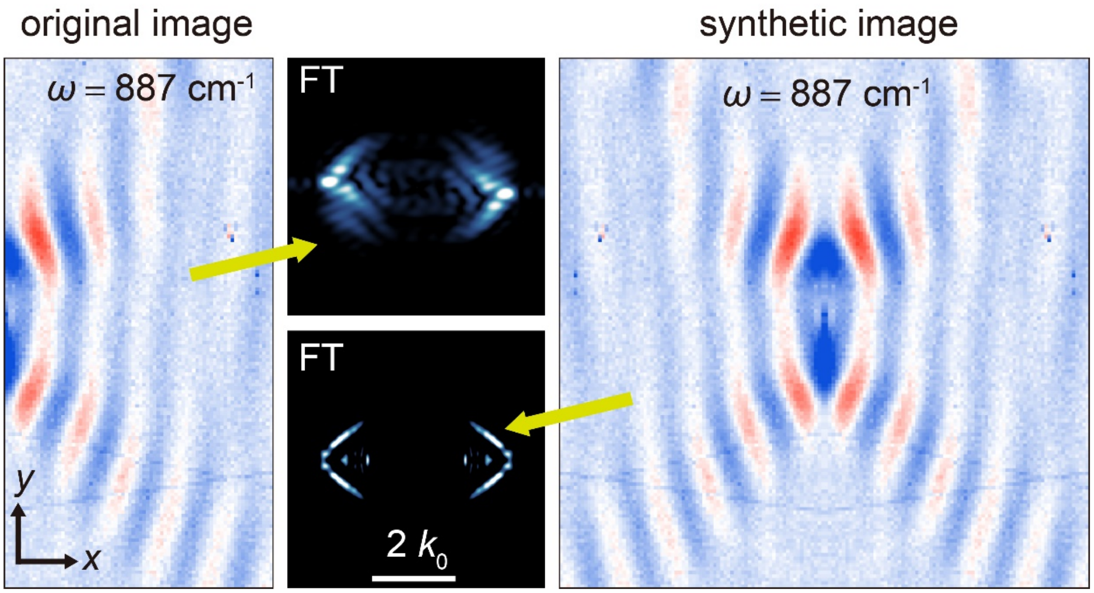

Figure S12. Analysis of experimental near-field images and corresponding IFC on calcite (θ= 23.3°) at ω = 887 cm^{-1}. The original experiment results exhibited a little difference for upper and bottom field distribution, thus rendering an asymmetric dispersion in *k*-space. These features are not the intrinsic properties of LPs, mainly from a little misalignment between direction of incident illumination and *x* direction. Synthetic image obtained by merging original and horizontally flipped mirror images, this procedure increases the number of images pixels for subsequent FT analysis with higher quality and also weak the faint influence from a little misalignment.

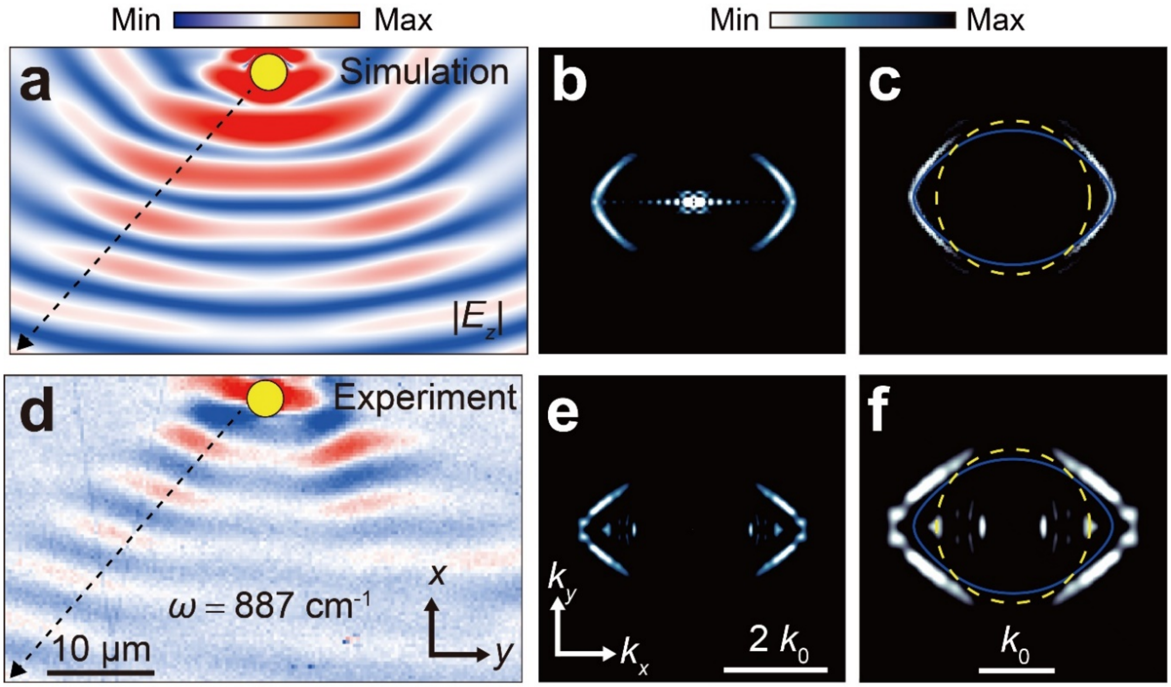

Figure S13. Experimental near-field images and simulation of disk-launched LPs. a. Simulation of disk-launched LPs at oblique illumination (a p-polarized wave with incident angle of 30° to calcite surface along x direction) at $\omega = 887\ \mathrm{cm}^{-1}$. d. The experimental results were recorded on calcite at ω = 887 cm^{-1}. b and e, The corresponding IFC of simulated disk-launched LPs in a and experimental results in d. The corrected IFC are shown in c and f. The yellow dashed line is free-space light cone and the blue solid line is the intrinsic in-plane dispersion of LPs.

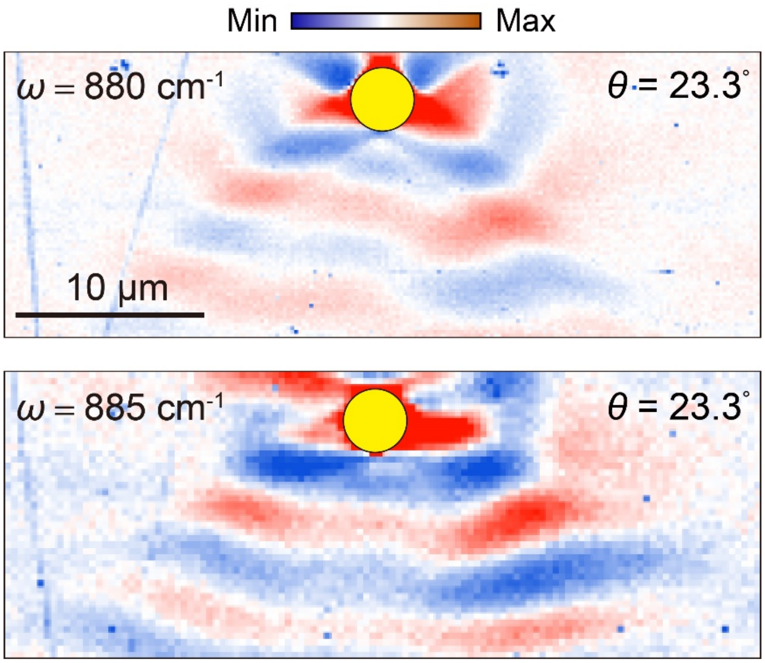

Figure S14. Real space imaging of LPs at other frequencies. The experimental results were recorded on calcite ($\theta = 23.3^{o}$) at ω = 880 cm^{-1} and ω = 885 cm^{-1}. The near-field distribution of disk-launched LPs also presented strong anisotropic propagation.

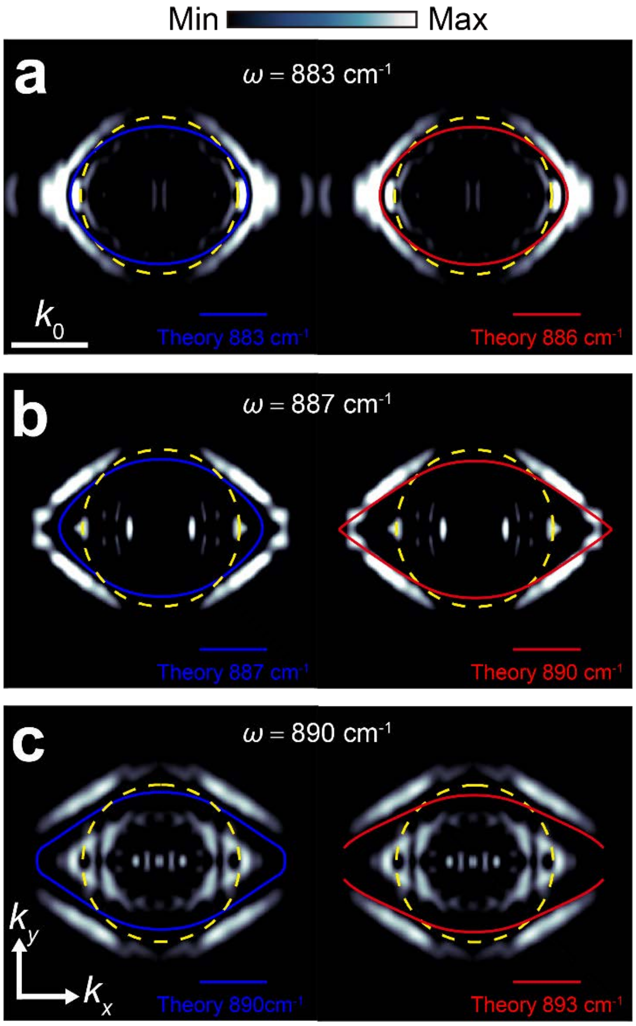

Figure S15. Frequency shift between the theory and experimental near-field results. a-c. IFC of LPs experimentally obtained at frequency a. $\omega = 883\text{cm}^{-1}$, b. $\omega = 887\text{cm}^{-1}$ and $\omega = 890\text{cm}^{-1}$. They overlay with the dispersions from the theory obtained at the same frequencies (blue lines) or at the shift frequencies (red lines) by adding $\Delta\omega = 3\text{cm}^{-1}$ on the frequencies taken in experiment. After adding the estimated frequency shift $\Delta\omega$, the red dispersions match slightly better the experimental results than the blue dispersions. The small deviation between the theory and the experiment might arise from the different phonon resonances or damping losses adopted in Lorentz model and the ones in the realistic calcite.

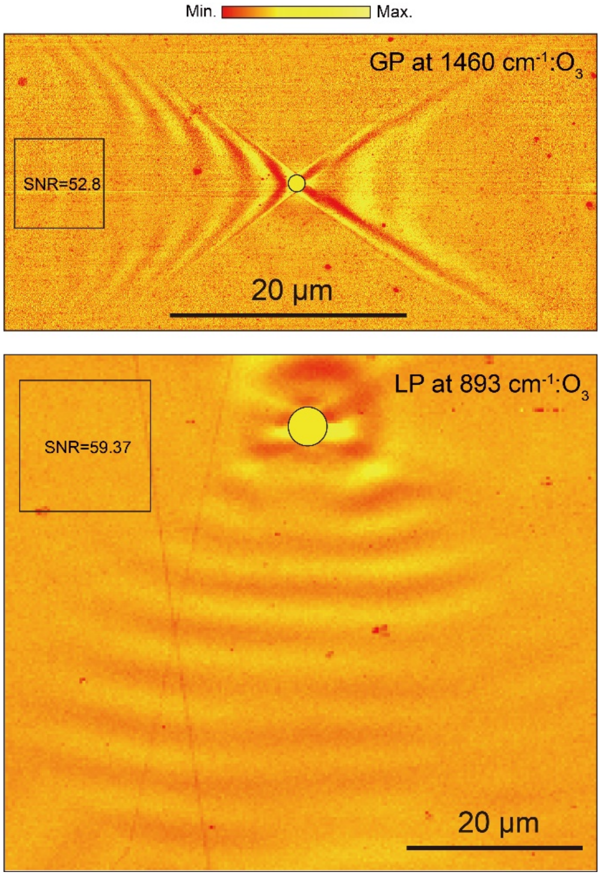

Figure S16: Compare the signal-to-noise ratio (SNR) between LPs and g-HPs: To compare the noise ratio of experimental results between LPs and g-HPs, we estimate the SNR (signal-to-noise ratio) respectively. Here we define the SNR as S_a/S_m, the S_a is the value of optical signal irregularities, S_m is the mean value in selected region, $S_a = \frac{1}{N}\sum_{n=1}^{N}(S_n - S_m)$. We get the similar SNR of LPs and g-HPs in the experiments which indicate a similar quality of measurements.

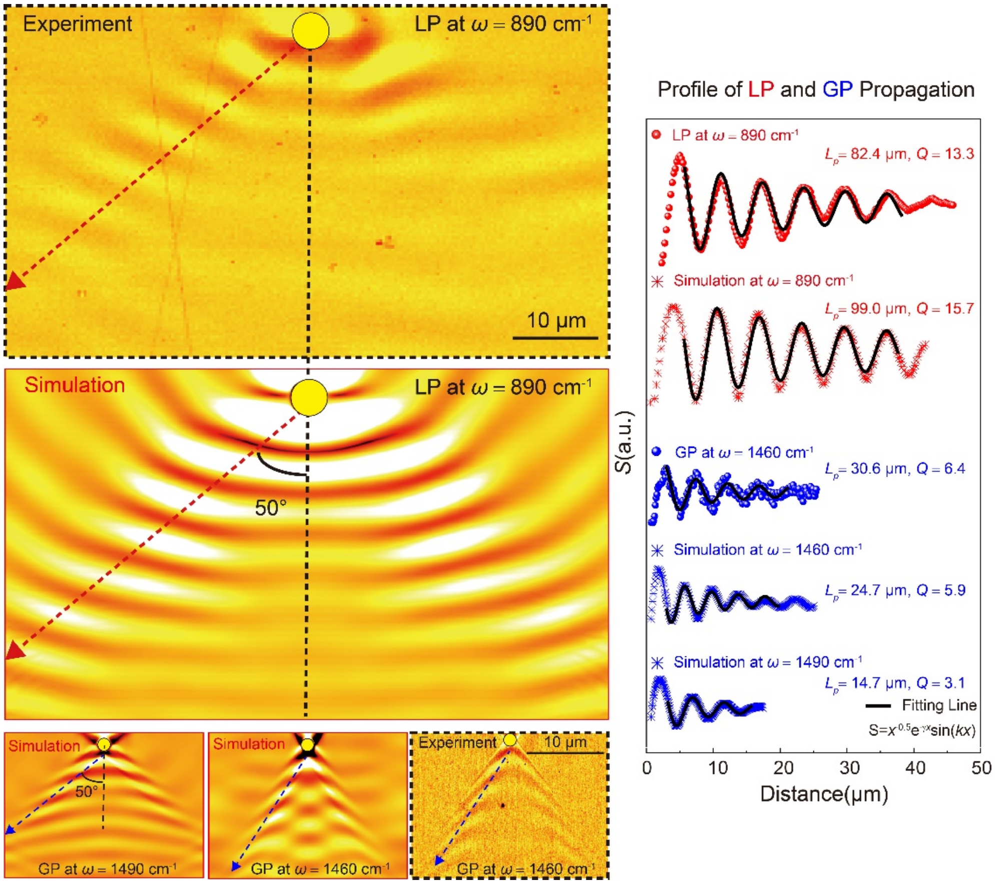

Figure S17: Compare the propagation length in real space between LPs and g-HPs: Left panel: LPs at ω= 890 cm^{-1} (Experiment), LPs at ω= 890 cm^{-1} (Simulation); g-HPs at ω= 1490 cm^{-1} (Simulation), g-HPs at ω= 1460 cm^{-1} (Simulation), g-HPs at ω= 1460 cm^{-1} (Experiment). The experiment and simulation of LPs and g-HPs both indicate a good agreement. Right panel: Line profiles of LPs and g-HPs propagation along the direction marked by the dashed arrow. By fitting the near-field profile of the LPs with a modified damped wave function： $\mathrm{Re}(E) = x^{-0.5} \bullet e^{-\gamma x} \bullet \sin(kx)$, where x is the distance away from the source, and we can estimate the Q value from k and γ. Considering the anisotropic propagation of LPs and g-HPs, we would like to compare the polariton propagation along a similar direction (defined by the half of open angles). It is noted that LPs at ω= 890 cm^{-1} and g-HPs at ω= 1490 cm^{-1} show a similar direction of polariton propagation. It is out of our accessible QCL source to measure the g-HPs at ω= 1490 cm^{-1}in real space. However, according to the simulation, anisotropic g-HPs at ω=1490 cm^{-1} is expected higher propagation loss than ω=1460 cm^{-1}(shown in the right panel). From the estimated propagation loss from both experiment and simulation results, we can witness a lower damping rate of LPs than g-HPs when weighing their anisotropic propagation.

b. Experiment for calcite with $\theta = 48.5^{\circ}$

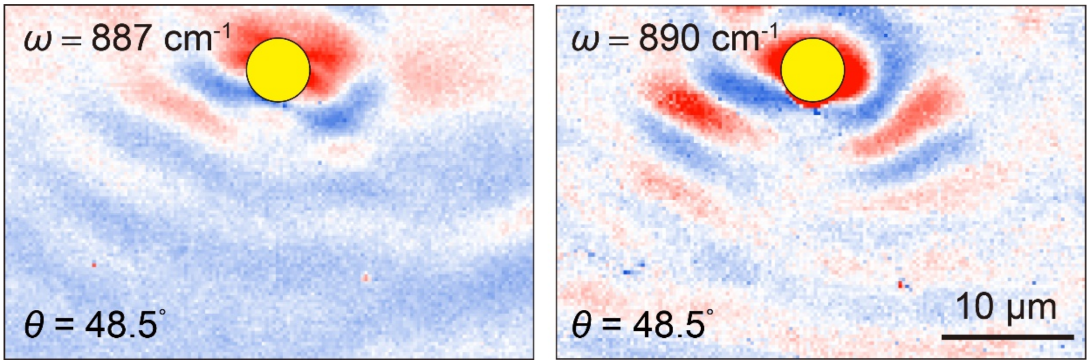

Figure S18. Real space imaging of LPs at $\theta = 48.5^{\circ}$ OA-orientations. The experimental results were recorded on calcite ($\theta = 48.5°$) at ω = 887 cm^{-1} and ω = 890 cm^{-1}. The directional propagation was also evidently seen, indicating the robust features of LPs for different OA orientations and the feasibility of OA engineer to tune the propagation of LPs at fixed frequency.

VII. Quantitative study of in-plane propagation directionality

We evaluate the directionalities of LPs and g-HPs respectively from their spatial energy flow through a circle with various radii r (Fig. S19(a,b)). The figure of merit (FOM) of in-plane propagation directionality is defined as follows:

$$\mathrm{FOM(r)} = \frac{1}{2\pi}\oint_{\mathrm{r}} d\phi \left|\tilde{E}\right|, \tag{21}$$

where $\left|\tilde{E}\right|$ is taken along the circle with radius r and normalized over the maximum as shown in Fig. S19 (c,d). Based on this definition, the range of FOM is between 0 and 1, therefore, the smaller FOM is, the more directional the in-plane propagation of modes is. As we show in Fig. S19(e), when $r/\lambda_0 < 1.75$, where λ_0 is the free space wavelength, the directionality of LPs is not as good as g-HPs. However, as r increases, LPs become more directional, but g-HPs is slightly less directional. when $r/\lambda_0 \geq 1.75$, LPs exhibit similar or even superior directionality than g-HPs do. The distinct behaviors of directionality between LPs and g-HPs can be explained by different orientation between maximum dissipation and major power flow: in g-HPs case, the direction of maximum dissipation follows the same direction as the one of their major energy flux. As a result, when g-HPs propagate away from the point source, their fields distribute more broadly in azimuthal direction (Fig. S19 (c)), therefore reducing their spatial directionality. However, in LPs case, the direction of maximum dissipation (which follow the direction of OA, as shown in Fig. S7(b)) is not in parallel with the direction of major energy flow, thus the width of LPs' in-plane power flow becomes narrower in space as they propagate away from the point source, as shown in Fig. S19(d).

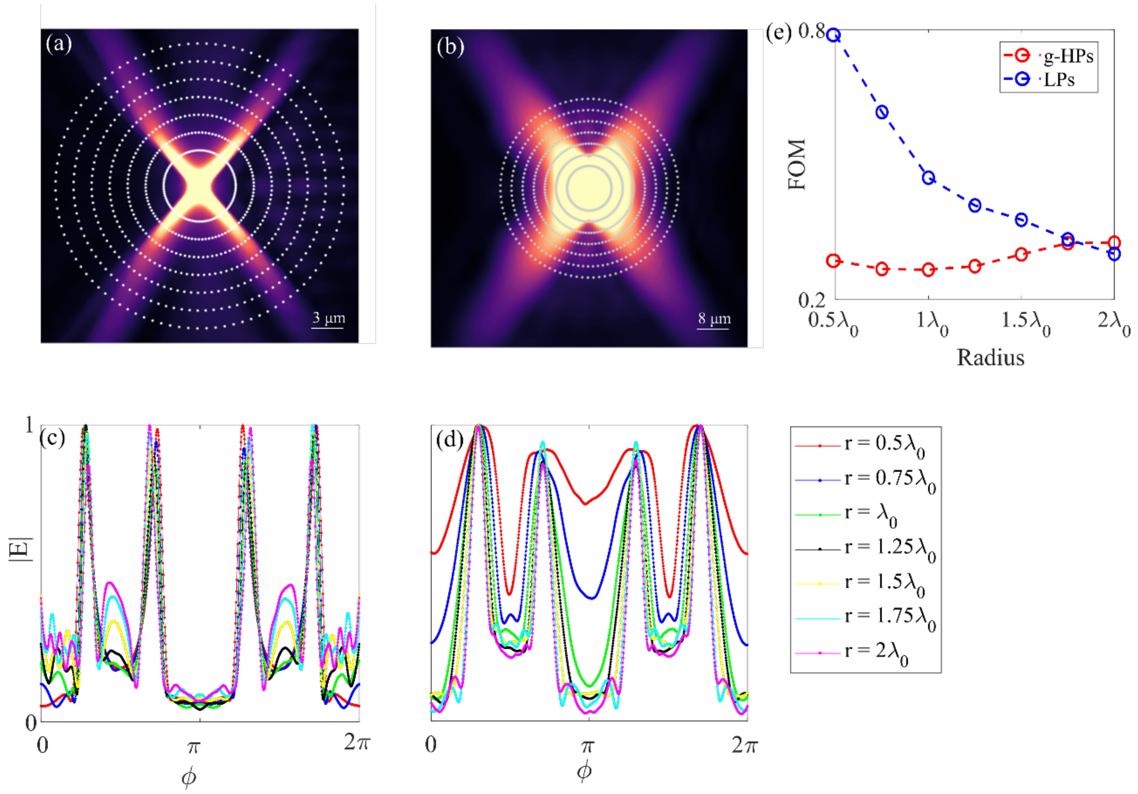

Figure S19: Quantify the directionality of in-plane propagation of LPs and g-HPs. (a,b) Electric field amplitudes of polaritons in real space showing directional propagation at frequency: (a) $\omega = 1470\text{cm}^{-1}$, corresponding to g-HPs and (b) $\omega = 890\text{cm}^{-1}$, corresponding to LPs. (c,d) Electric field amplitudes of polaritons vs azimuthal angle ϕ, where the fields are taken along various circles with radius r denoted by white dots in (a,b). (e) Figure of merit (FOM) of directionality vs radius of LPs and g-HPs.

VIII. Directionality comparison of far-field emission

Because of the in-plane directionality of hybridization between evanescent extraordinary wave and radiative ordinary wave, the excitation of LPs with a localized emitter radiates in calcite more directionally than hyperbolic bulk polaritons do. In Figure S20 we compare the radiation sustained by LPs and the one of hyperbolic bulk polaritons in calcite at different wavelengths, demonstrating different directional radiation response in the bulk of calcite. The fields are calculated for a small emitter placed at 200nm from the interface.

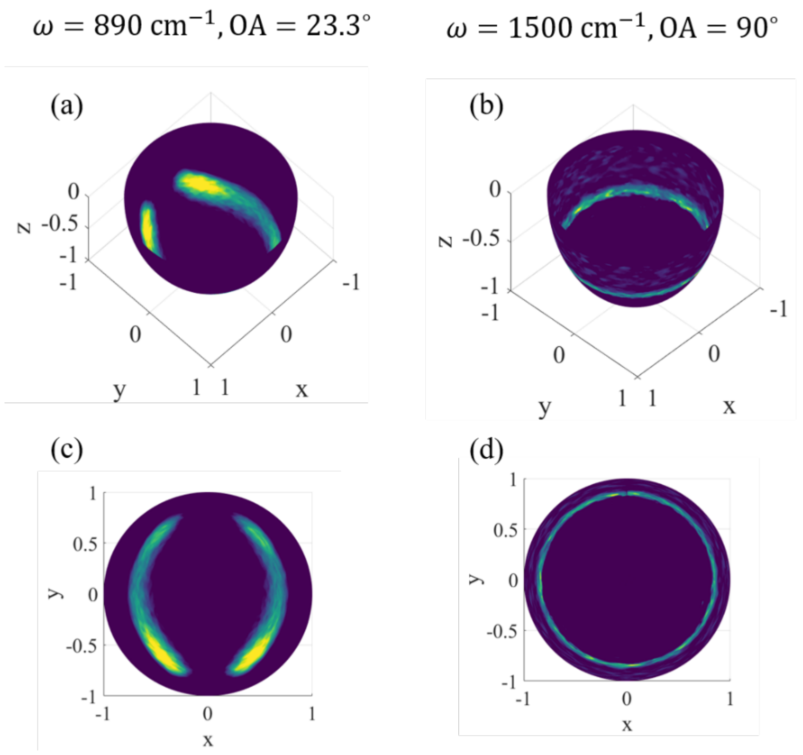

Figure S20: Compare the directionality of far-field emission between LPs and hyperbolic bulk polaritons. (a) 3D perspective view and (b) top view of time average energy density emission ⟨W⟩ of LPs on the half sphere inside calcite in which OA= 23.3° ; (c) 3D perspective view and (d) top view of time average energy density emission ⟨W⟩ of hyperbolic bulk polariton inside calcite in which OA= 90°. The point emitter is located 200nm above the interface of the material.

IX. Polariton life time and propagation losses

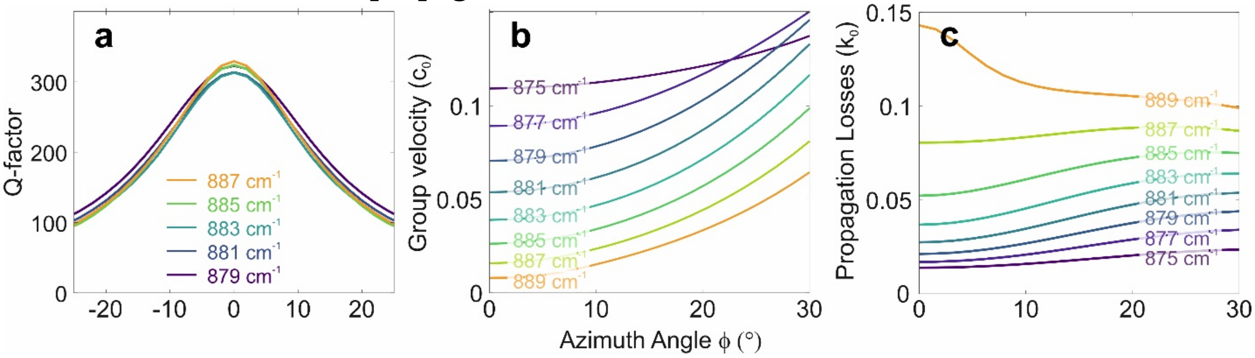

Figure S21. Azimuthal dependence of LP Q factor, group velocity and propagation losses. a. Simulated Q-factor of the spectral LP resonance as shown in Fig. 3i of the main text. b. Analytical LP group velocity. c. LP propagation losses, all calculated for (100) Calcite. Notable, the rapidly decreasing Q-factor, i.e. proportional to the inverse LP life time, is compensated by the increasing group velocity (b), resulting in nearly constant (azimuthally independent) propagation losses (c).

X. Experimental data for far-field probing

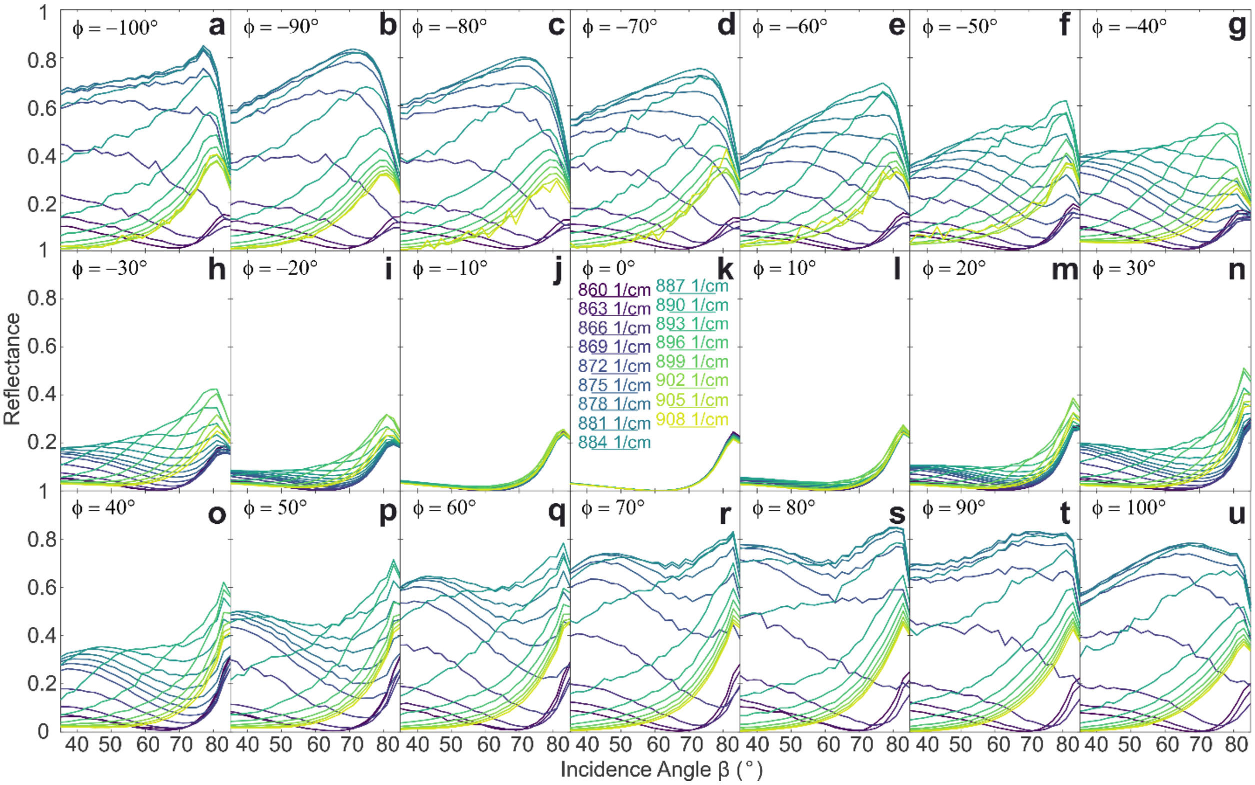

Figure S22. Incidence angle scans at various azimuth angles. a-u. Experimental data for far-field reflectance measurements. The (100) calcite sample has been positioned at different azimuthal angle orientations φ; at each of them, the angle of incidence β has been scanned at various FEL frequencies around the Reststrahlenband of calcite. Each panel (a-u) displays the reflectance scans at a fixed azimuthal angle for the considered frequencies. For every azimuthal orientation, we observed an abrupt decrease of the reflectivity at, on average, angle of incidence around 81°, which arises because of clipping of the FEL beam at the narrow projection of the sample area at large incidence angles, such only part of the beam is reflected at the sample. In order to ensure consistency among data sets taken at different azimuthal angles and laser alignment, a normalization technique has been implemented: The raw data set for each azimuthal angle are normalized by the maximum reflectance value at β = 81° obtained from the equivalent transfer matrix simulations. The result of this normalization procedure is shown here.

Reference

1 Ma, W. L. *et al.* Ghost hyperbolic surface polaritons in bulk anisotropic crystals. *Nature* **596**, 362–366 (2021).

2 Lampariello, P., Frezza, F. & Oliner, A. A. The Transition Region between Bound-Wave and Leaky-Wave Ranges for a Partially Dielectric-Loaded Open Guiding Structure. *Ieee T Microw Theory* **38**, 1831-1836 (1990).
